# Modulation of nutritional composition and aroma volatiles in cultivated pork fat by culture media supplementation

**DOI:** 10.3389/fnut.2025.1674183

**Published:** 2025-10-14

**Authors:** Natsu Sugama, Emily T. Lew, Camilo Riquelme-Guzmán, Di Sheng Lee, Xinxin Li, John S. K. Yuen, Taehwan Lim, Anson Kwan, Run Yi Liu, Yoshene A. Ma, Scott C. Frost, David L. Kaplan

**Affiliations:** ^1^Tufts University School of Engineering, Medford, MA, United States; ^2^Tufts University Cellular Agriculture Commercialization Laboratory, Boston, MA, United States; ^3^Deco Labs, Inc., Boston, MA, United States; ^4^Tufts University School of Arts and Sciences, Medford, MA, United States

**Keywords:** cellular agriculture, cultivated meat, fat, adipogenesis, flavor, aroma, sensory

## Abstract

Cultivated meat is emerging as a novel food source with the potential to contribute to a more sustainable and ethical food production system. However, limited research to date has explored the extent to which the nutrition and the aroma of such foods can be altered through cell culture conditions. Here, we aimed to modulate the aromatic volatile compounds in heated porcine cultivated fat cells by manipulating the media components while ensuring the preservation of robust fat differentiation. Using dynamic headspace gas chromatography–mass spectrometry (DHS-GC–MS), we demonstrated that supplementing cells with thiamine-HCl increased its intracellular concentration and promoted the production of 4-methyl-5-thiazoleethanol, contributing to milky aroma. Similarly, supplementation with L-methionine enhanced its intracellular concentration and increased the production of methional, a volatile compound with a potato-like aroma. Additionally, myoglobin significantly altered the volatile organic compound profile of cultivated fat. Notably, the concentration of *γ*-nonalactone, (E, E)-2,4-decadienal and 2-pentylfuran were increased, which contribute to a coconut-like, deep fat, fruity aroma, respectively, as well as elevated levels of other alcohols, aldehydes and furans. These findings highlight the potential of culture media formulations to modulate the aroma in cultivated fat production, a unique opportunity to optimize sensory features using this novel food production technology.

## Graphical abstract

Graphical overview of the methodology. Porcine dedifferentiated fat cells (pDFAT) were differentiated into adipocytes using adipogenesis media supplemented with aroma precursors. The cells were heated (cooked) and the resulting volatile compounds were analyzed using dynamic headspace gas chromatography–mass spectrometry (DHS-GC–MS).
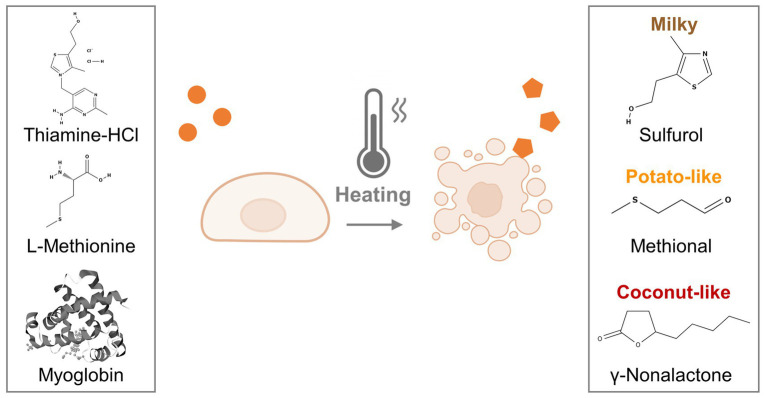

## Highlights

- Nutrient composition and aroma profiles of cultivated pork fat upon baking were modulated by cell culture media supplementation.
- Supplementing with thiamine-HCl, L-methionine, or myoglobin increased intracellular levels of thiamine or methionine and modulated the formation of aroma volatiles, enhancing characteristic odors such as milky, potato-like, and coconut-like notes.

## 1 Introduction

Cultivated meat has emerged as a promising technology to produce meat sustainably, with a significantly reduced risk of infectious diseases along with potentially improved nutrition (1, 2). A cornerstone for the success of this technology is flavor, which is a significant factor influencing consumer acceptability and purchasing decisions for meat products (3–6). Nevertheless, cultivated meat is still in its early stages of development, and many claims remain speculative rather than established facts (7). With respect to flavor, cultivated meat may differ substantially from conventional meat, particularly in its amino acid and nucleotide composition (8), however, research on its sensory characteristics remains limited (9). In terms of flavor, fat plays a crucial role in retaining aroma compounds and contributing to the persistence of scent (10, 11). Additionally, lipid oxidation products, in combination with Maillard compounds, produce a wide variety of aroma compounds in cooked meat (12–15).

Recently, several studies have reported on the aroma profiles of cultivated fat and muscle (16–18). For example, we have characterized volatile organic compounds (VOCs) released during the cooking of cultivated fat derived from porcine dedifferentiated fat cells (pDFAT) (16). Our study revealed the presence of fatty aldehydes such as pentanal, hexanal, octanal, and nonanal which contributed to fatty and buttery aromas. Sensory evaluation showed no statistical difference in response to the cooked cultivated pork fat when compared to traditional livestock-derived pork fat. Another study analyzed the VOCs of porcine adipocytes derived from porcine adipose-derived stem cells (ADSCs) cultured with scaffolds and found that both conventional and cultivated fat shared multiple common VOCs (17). A separate study revealed that porcine fibroblasts and myoblasts cultured in 10 and 15% serum-containing media exhibited significantly higher concentration of thiophenes which impart a meaty aroma than those cells maintained with 1% serum-containing media (18). These results suggested that cultivated meat and fat contain aroma volatiles similar to those found in livestock grown meat and fat, although some differences are present. Additionally, the media composition may influence the types and quantities of VOC profiles produced by baked cells.

In meat science, changing the composition of animal feed can modify VOC profiles, leading to changes in flavor (19–21). Diets with low protein and well-balanced essential amino acids significantly increased the level of 2-heptanone, which has a fruity smell, and 2,3-octanedione, which imparts the characteristic aroma of pork (21). Additionally, post-harvest treatment of cooked ham with thiamine can increase the concentration of 2-methyl-3-furanthiol and bis(2-methyl-3-furyl)-disulfide, which showed a significant difference in taste tests. These results demonstrated that the flavor of meat can be modified by adding certain nutrients to the diet of livestock. It is also possible to change the aroma during secondary processing after harvesting from the animal for processed meats like ham. However, to provoke these types of aroma changes in meat, 60 to 70 days of feeding is required (21, 22). Furthermore, the addition of aroma post-harvest requires secondary processes such as curing, and the presence of nitrites or nitrates used in processed meats can impact the changes in aroma (23). An important potential advantage of cultivated meat technology is in the ability to tailor aroma and nutritional content during cell cultivation due to the direct access of the media to the cells (24). Despite this potential, no research to date has specifically targeted the regulation of volatile aroma compounds in cultivated meat or fat through optimization of media composition.

The pathways for VOC generation in meat can be classified into the Maillard reaction, thiamine degradation, lipid oxidation, and Maillard-lipid interactions (25, 26). To address these pathways, three additives were studied: (1) thiamine plays a critical role as a coenzyme in carbohydrate metabolism and neural function, and its deficiency is associated with neurodegenerative disorders (27). In addition, during thermal degradation, thiamine generates aroma compounds such as furanthiols (28), thiophenes (29), and thiazoles (30), which contribute to meaty and nutty aromas characteristic of baked meat; (2) L-methionine is an essential amino acid involved in methylation reactions, antioxidant defense via glutathione synthesis, and hepatic function. Upon heating, it undergoes Strecker degradation to produce methional, a well-known aroma compound with a savory, potato-like odor that is commonly found in pork, beef, and chicken (31–35). (3) Myoglobin, which contributes to the generation of aroma compounds, as its iron content catalyzes lipid oxidation reactions (36). Additionally, its presence in plant-based meat increases the formation of lipid oxidation products during heating (37).

Here, we aimed to modulate the volatile compound profile of cultivated pork fat cells by manipulating media components. This approach offers a promising opportunity to leverage media formulation as a tool to enhance the sensory qualities of cultivated fat, thereby advancing applicability in food systems.

## 2 Results

### 2.1 Characterization of porcine dedifferentiated fat cells and optimization of growth and adipogenesis media

**Figure 1:**
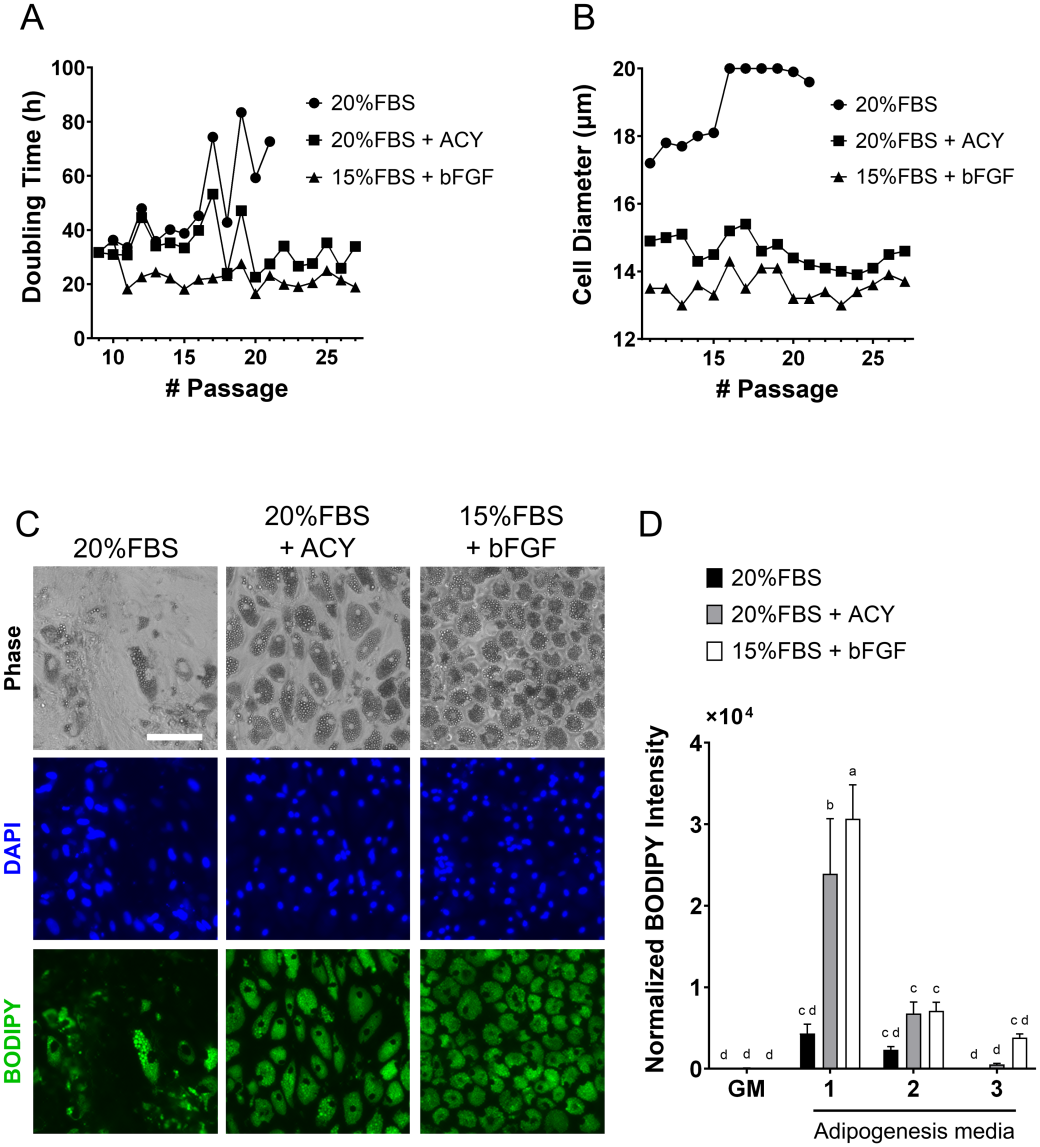
Characterization of pDFAT cells maintained under three different proliferation media, ‘20%FBS’, ‘20%FBS + ACY’ and ‘15%FBS + bFGF’, as well as three different adipogenesis media (1 to 3) to achieve rapid cell growth and maintaining adipogenic capability during continuous passage. **(A)** Hours per cell doubling. **(B)** Cell diameter. **(C)** Morphology of adipocytes maintained with three different proliferation media. Lipids were stained with BODIPY (p23). Scale bars, 100 μm. **(D)** Lipid quantification was performed using BODIPY staining. Average BODIPY integrated intensity was multiplied by BODIPY count and divided by the number of nuclei. GM refers to growth media. Adipogenesis was induced using three different adipogenesis media (Media 1, 2 and 3). n = 5 for each group. Statistical significance was determined using Two-way ANOVA with multiple comparisons. Different letters indicate statistically significant differences among groups (*p* < 0.05, Mann–Whitney test).

To achieve rapid cell proliferation, as well as the maintenance of adipogenic capability over multiple passages and efficient adipocyte differentiation, we evaluated proliferation and adipogenesis media using a pragmatic selection guided by previous reports (38–41), rather than through systematic optimization. Cells were cultured using three different growth media formulations which developed based on previous publications, here in after ‘20%FBS’, ‘20%FBS + ACY (A 83–01, CHIR99021 and Y-27632)’ and ‘15%FBS + bFGF (basic fibroblast growth factor)’, in (Supplementary Table S1). The results showed that cells cultured in 20%FBS experienced slower proliferation, with 72.7 h doubling time at passage 19, leading to the termination of this condition. In contrast, cells cultured with 20%FBS + ACY exhibited an average doubling time of 33.4 h, while those cultured with bFGF displayed the fastest growth, with an average doubling time of 22.8 h (Figure 1A). Cell diameters were also monitored across passages (Figure 1B). Cells cultured in medium containing 20%FBS reached an average diameter exceeding 20.0 μm (the maximum quantification limit), while those cultured in 20%FBS + ACY and 15%FBS + bFGF media maintained smaller diameters, with averages of 14.6 μm and 13.6 μm, respectively.

The optimal composition of adipogenesis media were studied. The three different adipogenesis media formulations were based on published media (42–44) (Supplementary Table S1), here in after referred to as “Media1, 2 and 3.” The morphology of the cells maintained in ‘20%FBS’, ‘20%FBS + ACY’ and ‘15%FBS + bFGF’ proliferation media and induced adipogenesis by Adipogenesis Media1 was stained with BODIPY (Figure 1C). Among the condition of proliferation media, ‘20%FBS’, ‘20%FBS + ACY’ or ‘15%FBS + bFGF’, and Adipogenesis Media1, 2 and 3 were tested, the combination of cells maintained in ‘15%FBS + bFGF’ proliferation media and induced to undergo adipogenesis using Media1 exhibited the highest lipid accumulation capacity compared to cells cultured under all other growth media conditions (Figure 1D). Therefore, the combination of proliferation media ‘15%FBS + bFGF’ and Adipogenesis Media1 was selected for subsequent experiments.

### 2.2 Analysis of cell proliferation and adipogenic efficiency with aroma precursor supplementation

**Figure 2:**
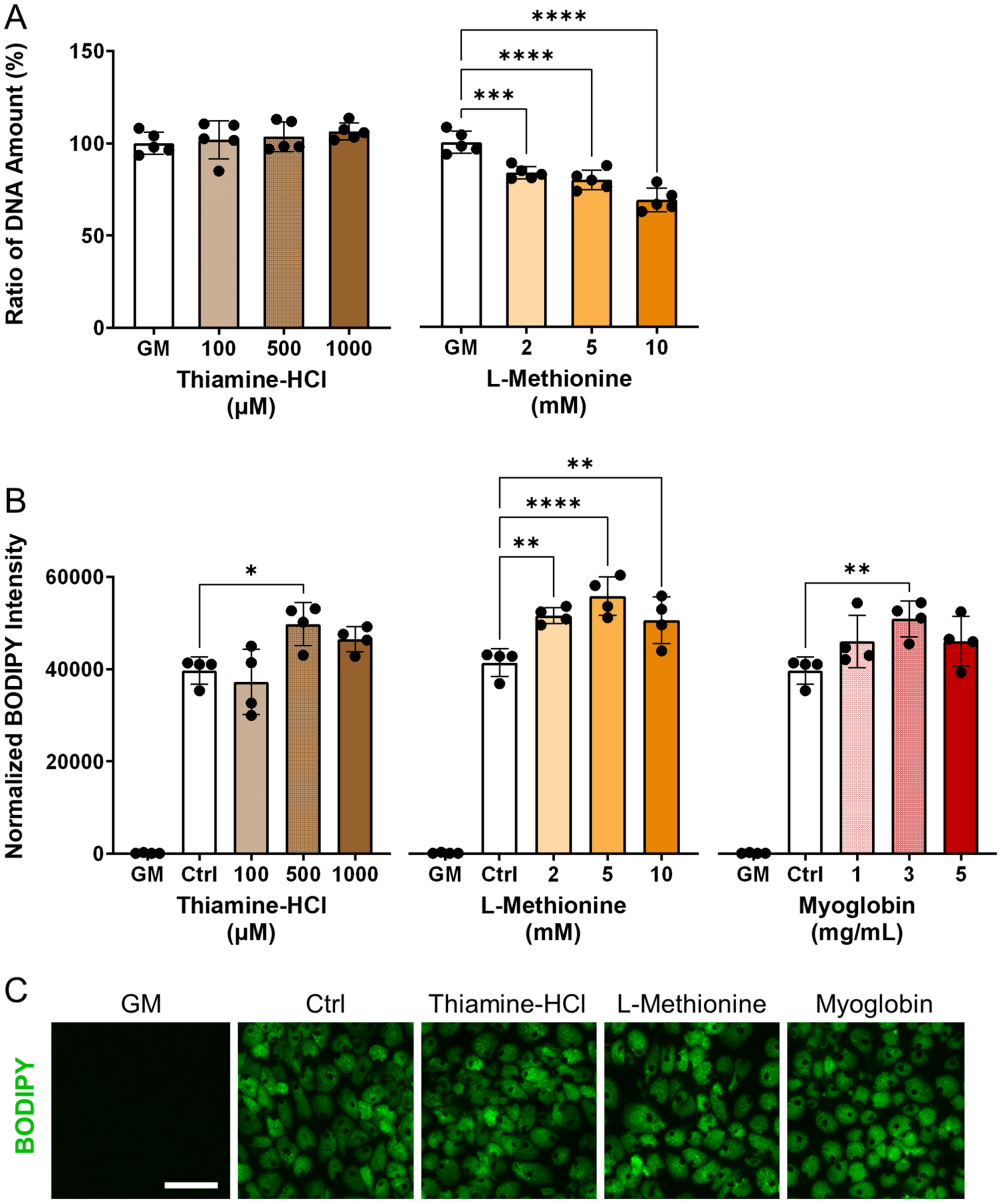
Effect of media supplements on proliferation and adipogenesis in pDFAT cells. **(A)** DNA quantification for pDFAT cells maintained with the proliferation media supplemented with different media additives (*n* = 5). The non-supplemented growth media condition (GM) was considered as 100%. **(B)** Lipid quantification of pDFAT-derived adipocytes treated with supplements during adipogenesis lipid accumulation period. Average BODIPY integrated intensity was multiplied by BODIPY count and divided by the number of nuclei. GM; cultured in proliferation media, Ctrl; cultured in adipogenesis media without supplementation. Statistical significance was determined using one-way ANOVA followed by Dunnett’s test (*, **, ***, **** denote *p* < 0.05, *p* < 0.01, *p* < 0.001 and *p* < 0.0001, respectively) compared to Ctrl. **(C)** Morphology of pDFAT-derived adipocytes cultured in adipogenesis media supplemented with 500 μM Thiamine-HCl, 5 mM L-Methionine or 3 mg/mL Myoglobin. Scale bars, 100 μm.

To investigate the effects of thiamine-HCl or L-methionine on cell proliferation, relative DNA amount was quantified (Figure 2A). Additionally, the effects of thiamine-HCl, L-methionine and myoglobin on lipid accumulation were assessed (Figures 2B, C). During the adipogenesis lipid accumulation period, supplementation with thiamine-HCl and L-methionine did not result in a decrease in lipid quantities. However, during the cell proliferation period, supplementation with L-methionine at concentrations above 2.0 mM reduced cell growth. Therefore, all supplements were added only during the adipogenesis lipid accumulation period since there was no beneficial effect of the supplementation on cell proliferation. Optimal concentrations, 500 μM thiamine-HCl, 5.0 mM L-methionine, and 3.0 mg/mL myoglobin were chosen for the supplementation for the rest of this work.

### 2.3 Metabolite analysis of cultivated fat before cooking

**Figure 3:**
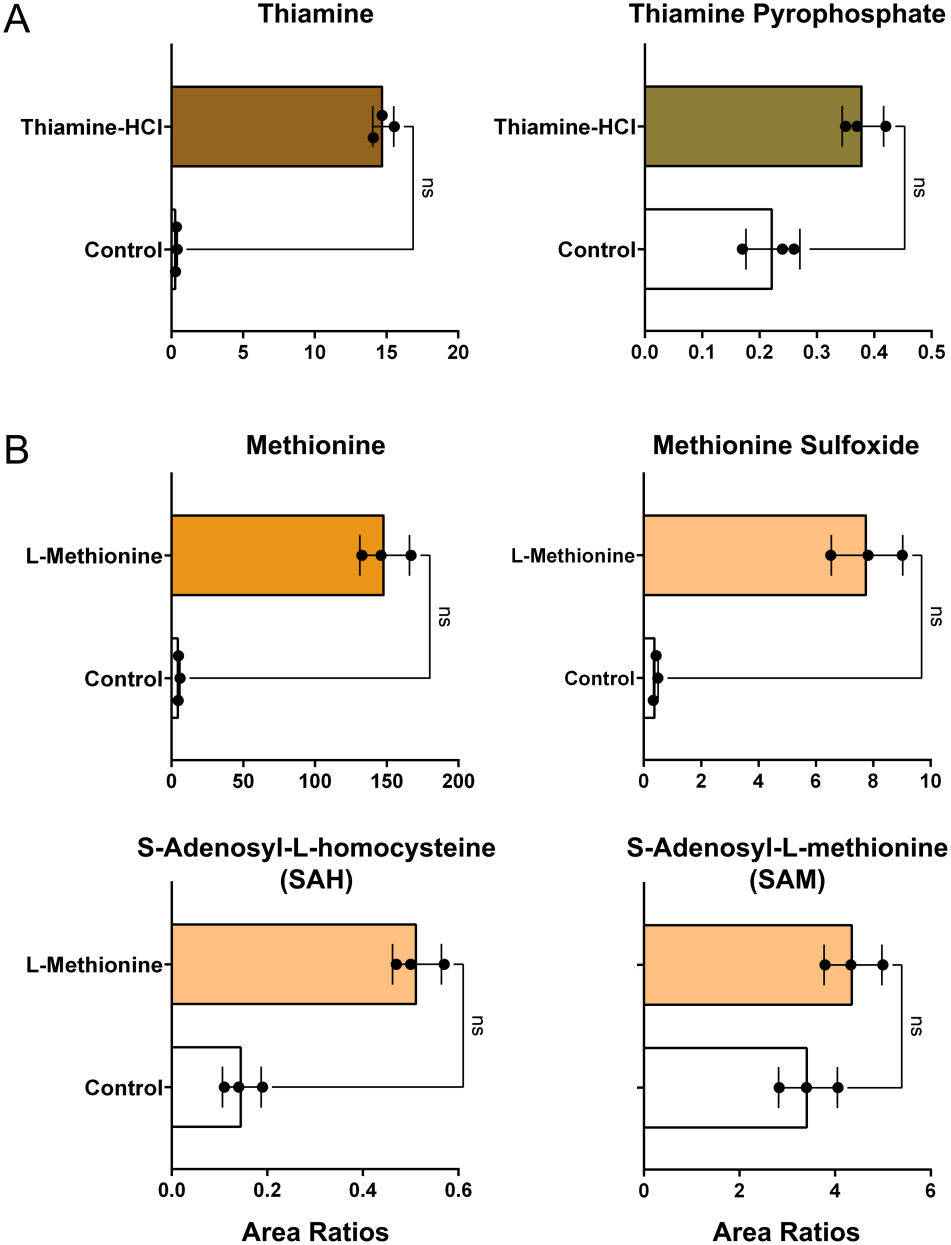
Relative levels of metabolites in cultivated fat. **(A)** Peak area ratios of thiamine and thiamine pyrophosphate in non-supplemented control and thiamine-HCl-supplemented cells prior to baking (mean±SD, *n* = 3). **(B)** Peak area ratios of methionine and its downstream intermediates in non-supplemented control and L-methionine-supplemented samples prior to baking (mean±SD, *n* = 3). Area ratios were calculated using the following formula: [{(Target Peak Area)/(Heavy Carbon-Labeled Phenylalanine or Methionine) divided by protein content measured by BCA assay. Statistical significance was assessed using the non-parametric Mann–Whitney test.

Relative levels of thiamine, thiamine pyrophosphate, methionine, methionine sulfoxide, S-adenosyl-L-homocysteine (SAH), and S-adenosyl-L-methionine (SAM) were quantified. No statistically significant differences were detected; however, a tendency toward increased thiamine pyrophosphate, a downstream metabolite of thiamine, was observed (Figure 3A). Similarly, levels of SAH, an immediate downstream metabolite, and SAM, a subsequent downstream metabolite of L-methionine, were not significantly different but both showed an increasing trend relative to the non-supplemented control (Figure 3B). Methionine sulfoxide, an oxidative product of L-methionine (45), also exhibited a tendency toward elevation. The metabolic pathways of thiamine and L-methionine in pig (*Sus scrofa*) were confirmed using the KEGG database (ssc00730, ssc00270).

### 2.4 Fatty acid analysis of harvested fat before cooking

**Figure 4:**
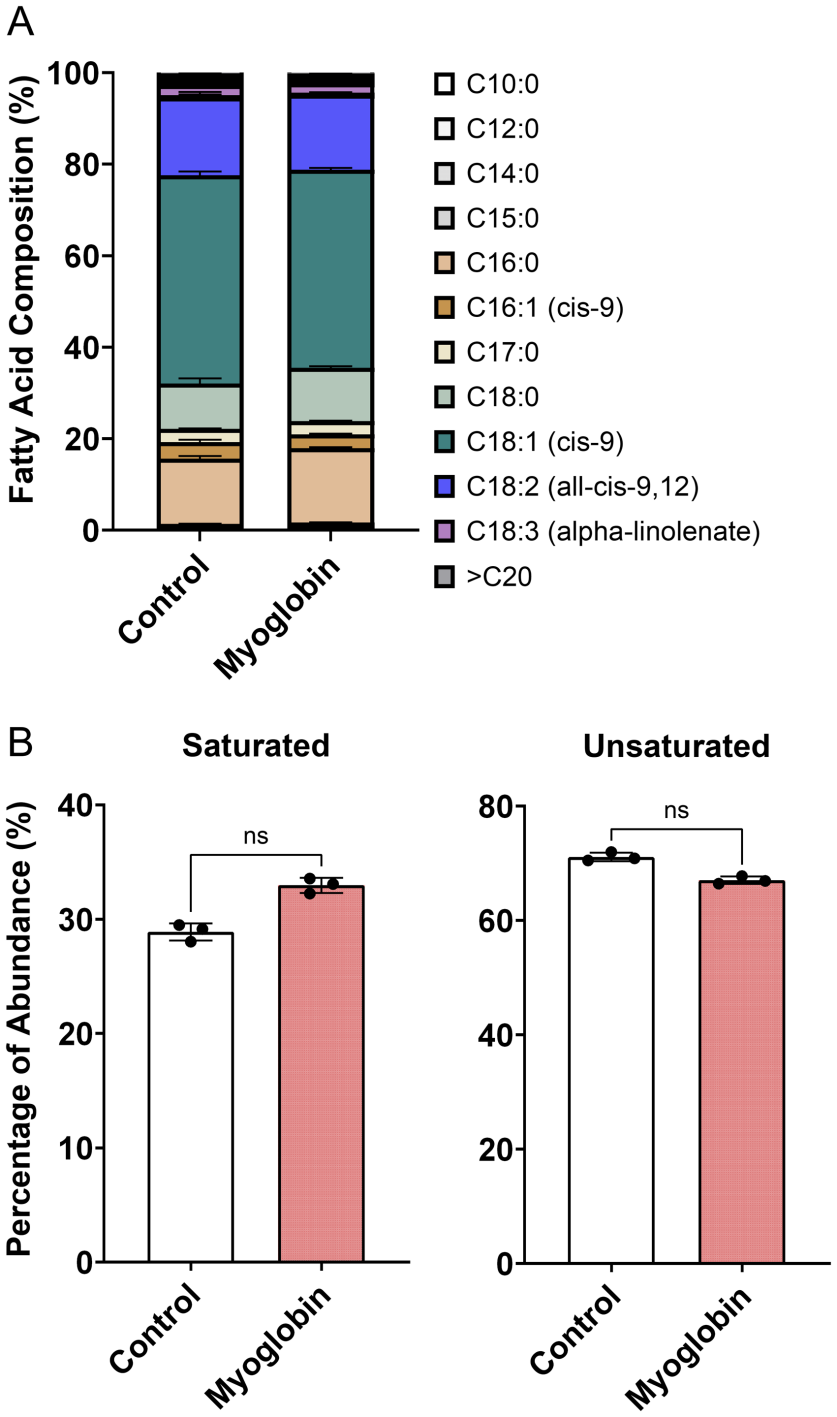
Fatty acid profile of cultivated fat. **(A)** Fatty acid composition between non-treated control and myoglobin, prior to baking (mean±SD, *n* = 3). **(B)** Percentage of total saturated and unsaturated fatty acids. Statistical significance was assessed using the non-parametric Mann–Whitney test.

To determine the specific types of fatty acids accumulated as triglycerides or phospholipids in cultivated fat, fatty acid analysis was performed. Additionally, the fatty acid profiles of both non-supplemented and myoglobin-supplemented cultivated fat were analyzed prior to baking, as the iron in myoglobin could potentially influence the composition. The results showed that the fatty acid composition of the non-treated samples consisted of 45.6% C18:1 (cis-9), 17.1% C18:2 (all-cis-9,12), 14.2% C16:0, and 9.89% C18:0, as the top four fatty acids identified (Figure 4A). There was no statistically significant difference between non-supplemented and myoglobin-treated cultivated fat in either unsaturated or saturated fatty acid content (Figure 4B).

### 2.5 Impact of aroma precursor supplementation on the concentration and profiles of volatile compounds from cells

**Figure 5:**
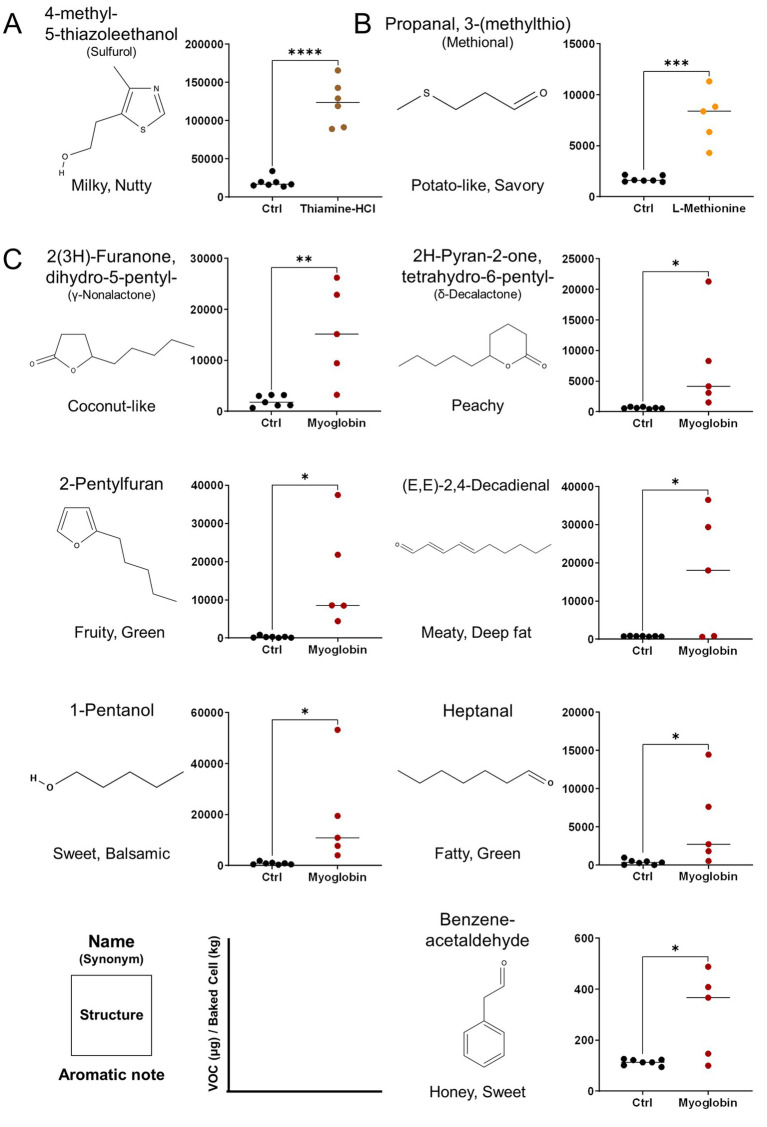
Major volatile organic compounds (VOCs) provoked by media supplementations and detected by DHS-GC–MS derived from pDFAT-derived adipocytes upon baking. VOCs altered under **(A)** 500 μM of Thiamine-HCl, **(B)** 5 mM of L-Methionine, **(C)** 3 mg/mL Myoglobin-supplemented conditions. VOCs were quantified by normalizing peak areas to two internal standards and converting the values to mass using authentic standard curves. The resulting concentrations were further normalized to cell mass after baking. ‘Ctrl’ indicates the non-supplemented cell condition. Replicates 5 to 7 include biological triplicates. Statistical significance was determined using unpaired t-test (*, **, ***, **** denote *p* < 0.05, *p* < 0.01, *p* < 0.001 and *p* < 0.0001, respectively). Compounds were identified by retention index (RI) and Mass Spec referencing NIST17 compared with authentic standards.

**Figure 6:**
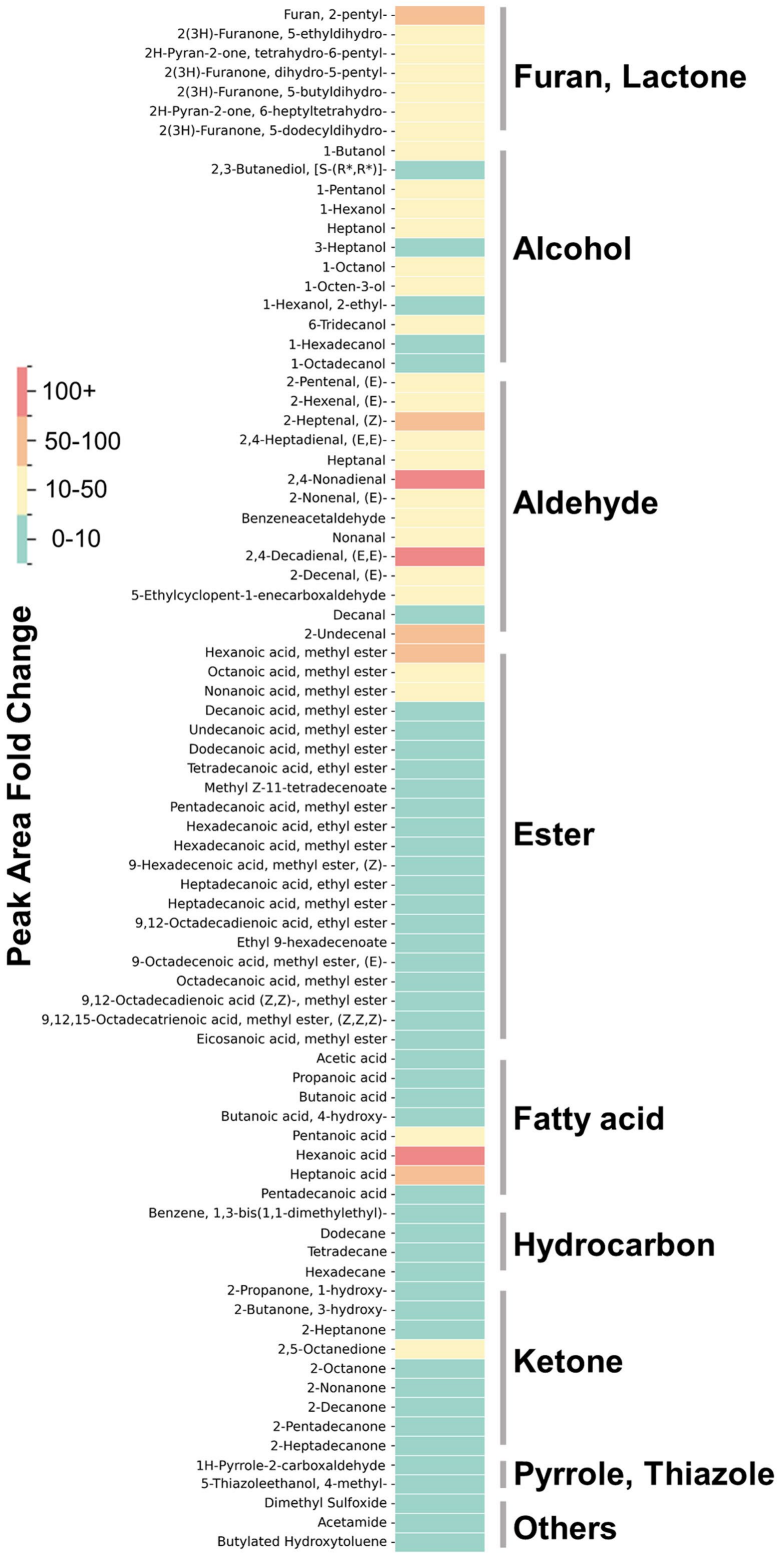
The fold change in the normalized peak area of VOCs found in myoglobin-supplemented cultivated fat, compared to non-supplemented control cells. The color intensity represents the value of the fold changes. All VOCs are tentatively identified except for VOCs shown in Figure 3.

The VOCs produced upon the addition of aroma precursors to the medium and subsequent heating/cooking were analyzed using DHS-GC–MS. The peaks of all compounds identified through deconvolution, were normalized using the internal standards and cell mass, described in ‘GC/MS data processing’ were represented in dot plots. Thiamine-HCl significantly induced the generation of 4-methyl-5-thiazoleethanol (sulfurol), milky aroma compound which derived from thiamine degradation (46, 47) (Figure 5A). L-methionine promoted the formation of methional, a well-known potato-like aroma compound (Figure 5B). The addition of myoglobin caused significant changes to the VOC profile, beginning with the formation of *γ*-nonalactone, (E, E)-2,4-decadienal, 2-pentylfuran, *δ*-decalactone, and benzeneacetaldehyde, which are responsible for the coconut-like, deep fat, fruity, peachy, and honey aroma of meat (48–51), while increasing heptanal and 1-pentanol which imparts green, fatty aroma (52, 53) (Figure 5C). Furthermore, myoglobin enhanced the production of various lipid degradation products, including aldehydes, alcohols, furans and some of ketones, fatty acids, and hydrocarbons, and phenolic derivatives. Some of the esters also showed an increase; however, the average fold change was not as significant compared to that of aldehydes, alcohols, and furans (Table 1). No significant changes were observed in the levels of pyrroles and thiazoles (Figure 6). All compounds and peaks found in the non-supplemented controls, along with those supplemented with thiamine-HCl, L-methionine and myoglobin are shown in (Tables 1–3).

**Table 1:** All the volatile organic compounds (VOCs) found in 120 °C heated cultivated fat maintained with non-supplemented media (Control) and supplemented with 3 mg/mL myoglobin.

		Normalized peak area^a^ (Average ± SD)			RI^c^		m/z (actual)			m/z (NIST)			
Name	CAS#	Control	Myoglobin	*p* value^b^	actual	ref	1	2	3	1	2	3	Identification
2(3H)-Furanone, dihydro-5-pentyl-	104–61-0	82,376 ± 68,085	835,894 ± 521,297	0.003190**	1993	1990	85	56	55	85	56	55	RI (standard), MS
Heptanal	111–71-7	12,790 ± 7,058	189,062 ± 139,903	0.006819**	1173	1171	70	44	43	70	44	43	RI (standard), MS
2-Pentylfuran	3777-69-3	13,615 ± 12,869	752,355 ± 633,427	0.010363*	1216	1215	81	138	82	81	138	82	RI (standard), MS
2H-Pyran-2-one, tetrahydro-6-pentyl-	705–86-2	16,414 ± 12,123	273,899 ± 231,899	0.013525*	2159	2151	99	71	70	99	71	70	RI (standard), MS
2,4-Decadienal, (E, E)-	25152–84-5	2,315 ± 2,561	501,819 ± 501,452	0.022709*	1789	1779	81	41	67	81	41	67	RI (standard), MS
1-Pentanol	71–41-0	13,376 ± 9,743	343,715 ± 361,323	0.033252*	1245	1255	42	55	70	42	55	70	RI (standard), MS
Benzeneacetaldehyde	122–78-1	4,516 ± 4,883	56,136 ± 43,618	0.010054*	1603	1606	91	120	92	91	120	92	RI (standard), MS
2-Octanone	111–13-7	16,546 ± 10,021	56,885 ± 13,573	0.000141***	1273	1278	58	43	71	43	58	41	RI (literature), MS
Benzene, 1,3-bis(1,1-dimethylethyl)-	1014-60-4	137,199 ± 21,572	76,582 ± 20,201	0.000603***	1411	1420	175	57	190	175	57	41	RI (literature), MS
3-Heptanol	589–82-2	6,262 ± 1999	2,243 ± 1,207	0.002618**	1289	1290	59	69	87	59	69	41	RI (literature), MS
2-Heptanone	110–43-0	67,869 ± 54,357	331,333 ± 177,990	0.003823**	1172	1178	43	58	71	43	58	27	RI (literature), MS
2,5-Octanedione	3214-41-3	3,475 ± 2,475	133,667 ± 99,257	0.005355**	1312	1319	43	99	71	43	71	99	RI (literature), MS
Hexadecane	544–76-3	25,761 ± 5,146	75,894 ± 39,826	0.007274**	1595	1600	57	71	43	57	43	71	RI (literature), MS
Undecanoic acid, methyl ester	1731-86-8	4,317 ± 5,207	28,428 ± 19,591	0.010155*	1683	1703	74	87	57	74	87	55	RI (literature), MS
Nonanoic acid, methyl ester	1731-84-6	1747 ± 828	75,160 ± 65,283	0.012543*	1478	1481	74	87	43	74	87	55	RI (literature), MS
5-Ethylcyclopent-1-enecarboxaldehyde	36431–60-4	2,172 ± 1,602	55,341 ± 47,905	0.013468*	1389	1399	67	95	124	124	95	67	RI (literature), MS
2H-Pyran-2-one, 6-heptyltetrahydro-	713–95-1	6,049 ± 4,995	68,461 ± 58,289	0.016512*	2388	2386	99	71	55	99	71	41	RI (literature), MS
Hexanoic acid	142–62-1	23,389 ± 21,096	2,730,517 ± 2,568,823	0.017382*	1826	1825	60	73	87	60	73	41	RI (literature), MS
Hexanoic acid, methyl ester	106–70-7	891 ± 563	86,083 ± 82,621	0.019310*	1177	1178	74	43	87	74	87	43	RI (literature), MS
Nonanal	124–19-6	35,540 ± 22,845	414,630 ± 372,498	0.020819*	1377	1379	57	56	41	57	41	43	RI (literature), MS
2(3H)-Furanone, 5-dodecyldihydro-	730–46-1	3,778 ± 1,324	51,289 ± 46,992	0.021248*	2796	2810	85	55	83	85	55	43	RI (literature), MS
2-Hexenal, (E)-	6728-26-3	1,411 ± 591	27,811 ± 26,410	0.022378*	1198	1196	69	41	55	41	42	39	RI (literature), MS
2-Undecenal	2463-77-6	2,727 ± 1914	258,985 ± 261,631	0.024533*	1732	1740	70	57	83	70	41	57	RI (literature), MS
2,4-Nonadienal	6750-03-4	889 ± 425	107,790 ± 113,224	0.028889*	1674	1681	81	41	67	81	41	27	RI (literature), MS
2-Butanone, 3-hydroxy-	513–86-0	266,483 ± 292,356	1,355,584 ± 1,150,513	0.034807*	1266	1265	45	43	88	45	43	88	RI (literature), MS
1-Octanol	111–87-5	9,176 ± 4,837	156,570 ± 166,187	0.037737*	1547	1554	56	55	69	56	55	41	RI (literature), MS
2-Heptenal, (Z)-	57266–86-1	3,003 ± 1,531	182,745 ± 206,055	0.040269*	1306	1318	83	41	55	41	27	55	RI (literature), MS
Octanoic acid, methyl ester	111–11-5	2,778 ± 964	28,400 ± 29,758	0.042538*	1379	1380	74	87	43	74	87	43	RI (literature), MS
6-Tridecanol	5770-03-6	924 ± 1,205	38,338 ± 43,739	0.043620*	1871	1865	83	69	55	55	69	83	RI (literature), MS
2-Pentadecanone	2345-28-0	19,339 ± 5,829	112,816 ± 109,555	0.044320*	2003	2002	58	43	59	58	59	43	RI (literature), MS
2(3H)-Furanone, 5-ethyldihydro-	695–06-7	9,395 ± 9,052	195,710 ± 220,808	0.046128*	1666	1665	85	57	56	85	29	56	RI (literature), MS
1-Hexanol	111–27-3	4,269 ± 3,034	137,340 ± 159,065	0.047497*	1347	1356	56	55	43	56	43	41	RI (literature), MS
2-Propanone, 1-hydroxy-	116–09-6	39,815 ± 13,317	138,159 ± 118,562	0.050786ns	1278	1275	43	74	42	43	31	74	RI (literature), MS
2-Heptadecanone	2922-51-2	10,049 ± 4,181	54,913 ± 55,589	0.055158ns	2213	2218	58	59	43	58	43	59	RI (literature), MS
1-Octadecanol	112–92-5	10,495 ± 2,160	101,085 ± 114,765	0.058929ns	2571	2570	83	97	69	43	83	55	RI (literature), MS
1-Hexadecanol	36653–82-4	17,368 ± 6,265	85,087 ± 88,119	0.065572ns	2363	2379	83	97	69	55	69	83	RI (literature), MS
1-Butanol	71–36-3	1,219 ± 904	15,516 ± 18,926	0.069059ns	1147	1150	56	41	43	56	31	41	RI (literature), MS
1-Octen-3-ol	3391-86-4	9,219 ± 5,086	99,784 ± 120,486	0.070152ns	1440	1445	57	43	45	57	43	72	RI (literature), MS
Decanoic acid, methyl ester	110–42-9	21,559 ± 28,721	57,752 ± 33,721	0.072700ns	1580	1583	74	87	143	74	87	55	RI (literature), MS
2-Nonenal, (E)-	18829–56-6	3,323 ± 1,349	126,984 ± 173,543	0.083248ns	1518	1519	70	55	83	43	55	70	RI (literature), MS
Butanoic acid, 4-hydroxy-	591–81-1	29,212 ± 13,897	99,181 ± 97,309	0.084839ns	1587	1601	42	86	56	42	41	86	RI (literature), MS
Hexadecanoic acid, ethyl ester	628–97-7	30,147 ± 32,156	144,002 ± 160,078	0.091763ns	2241	2243	88	101	157	88	101	43	RI (literature), MS
2,4-Heptadienal, (E, E)-	4313–03-5	1895 ± 992	62,246 ± 88,904	0.096717ns	1468	1463	81	110	53	81	110	41	RI (literature), MS
Methyl Z-11-tetradecenoate	124–10-7	11,733 ± 9,088	3,947 ± 3,048	0.098487ns	2028	2032	55	74	69	55	41	69	RI (literature), MS
Decanal	112–31-2	740 ± 329	7,197 ± 10,204	0.118540ns	1490	1498	57	55	43	43	41	57	RI (literature), MS
Heptanol	53535–33-4	5,638 ± 3,545	99,607 ± 150,876	0.123657ns	1446	1443	70	56	55	70	56	43	RI (literature), MS
Tetradecane	629–59-4	78,049 ± 21,807	107,459 ± 42,988	0.147673ns	1397	1400	57	71	43	57	43	71	RI (literature), MS
2(3H)-Furanone, 5-butyldihydro-	104–50-7	5,768 ± 6,138	106,683 ± 174,700	0.150201ns	1879	1878	85	57	56	85	29	56	RI (literature), MS
1-Hexanol, 2-ethyl-	104–76-7	12,255 ± 7,327	40,533 ± 48,966	0.156063ns	1478	1480	57	70	83	57	41	43	RI (literature), MS
9,12-Octadecadienoic acid, ethyl ester	7619-08-1	9,713 ± 11,443	28,148 ± 30,092	0.164608ns	2508	2515	81	67	95	67	81	55	RI (literature), MS
Dodecanoic acid, methyl ester	111–82-0	25,342 ± 29,784	55,076 ± 40,424	0.171086ns	1786	1770	74	87	43	74	87	41	RI (literature), MS
2-Pentenal, (E)-	1576-87-0	1,084 ± 1,022	12,512 ± 21,065	0.174345ns	1121	1124	55	83	84	55	84	83	RI (literature), MS
Dimethyl Sulfoxide	67–68-5	137,423 ± 72,663	323,042 ± 338,927	0.183101ns	1557	1553	63	78	61	63	78	45	RI (literature), MS
Butylated Hydroxytoluene	128–37-0	29,787 ± 36,643	5,993 ± 2,211	0.183228ns	1888	1902	205	220	206	205	220	57	RI (literature), MS
Pentadecanoic acid	1002-84-2	4,182 ± 4,692	17,745 ± 25,666	0.193944ns	2813	2819	73	60	43	73	43	60	RI (literature), MS
Tetradecanoic acid, ethyl ester	124–06-1	5,709 ± 6,549	17,592 ± 23,033	0.217667ns	2035	2040	88	101	55	88	101	43	RI (literature), MS
Heptadecanoic acid, ethyl ester	14010–23-2	2,935 ± 3,528	7,738 ± 9,005	0.223233ns	2345	2340	88	101	89	88	101	43	RI (literature), MS
2-Decenal, (E)-	3913-81-3	4,772 ± 2,510	191,888 ± 398,161	0.233190ns	1619	1616	70	55	43	43	41	55	RI (literature), MS
Heptanoic acid	111–14-8	463 ± 351	25,467 ± 54,269	0.241822ns	1951	1952	60	73	43	60	73	43	RI (literature), MS
Ethyl 9-hexadecenoate	54546–22-4	6,821 ± 6,200	13,522 ± 12,775	0.251321ns	2263	2267	55	88	84	55	88	69	RI (literature), MS
Pentanoic acid	109–52-4	1837 ± 1,199	91,217 ± 202,732	0.261352ns	1715	1712	60	73	41	60	73	41	RI (literature), MS
9,12,15-Octadecatrienoic acid, methyl ester, (Z, Z, Z)-	301–00-8	59,649 ± 66,034	26,617 ± 17,869	0.306799ns	2536	2550	79	95	67	79	67	95	RI (literature), MS
2-Nonanone	821–55-6	85,993 ± 106,850	158,430 ± 138,847	0.329436ns	1378	1379	58	43	71	43	58	41	RI (literature), MS
Acetamide	60–35-5	99,751 ± 50,533	144,417 ± 100,850	0.332076ns	1744	1764	59	44	43	59	44	43	RI (literature), MS
Hexadecanoic acid, methyl ester	112–39-0	1,004,330 ± 839,580	1,529,751 ± 951,396	0.335041ns	2201	2207	74	87	143	74	87	43	RI (literature), MS
2-Decanone	693–54-9	87,744 ± 106,860	171,055 ± 182,513	0.340118ns	1478	1476	58	43	71	58	43	71	RI (literature), MS
5-Thiazoleethanol, 4-methyl-	137–00-8	26,701 ± 12,560	16,913 ± 21,859	0.346100ns	2268	2275	112	143	113	112	113	143	RI (literature), MS
Butanoic acid	107–92-6	3,134 ± 2,639	13,924 ± 29,506	0.349439ns	1667	1663	60	73	42	60	73	41	RI (literature), MS
9-Hexadecenoic acid, methyl ester, (Z)-	1120-25-8	331,560 ± 301,167	216,426 ± 152,022	0.453857ns	2224	2242	55	69	74	55	69	74	RI (literature), MS
1H-Pyrrole-2-carboxaldehyde	1003-29-8	4,991 ± 5,037	7,538 ± 6,890	0.474269ns	1978	1978	95	94	66	95	94	66	RI (literature), MS
9,12-Octadecadienoic acid (Z, Z)-, methyl ester	112–63-0	7,804 ± 8,341	5,041 ± 1986	0.489845ns	2480	2488	81	55	67	67	81	95	RI (literature), MS
2,3-Butanediol, [S-(R*, R*)]-	19132–06-0	171,390 ± 76,733	126,696 ± 159,226	0.528643ns	1564	1544	45	57	43	45	43	29	RI (literature), MS
Heptadecanoic acid, methyl ester	1731-92-6	157,103 ± 206,241	105,465 ± 105,341	0.621451ns	2304	2307	74	87	143	74	87	43	RI (literature), MS
Dodecane	112–40-3	11,939 ± 11,224	8,966 ± 8,143	0.626289ns	1196	1199	57	71	43	57	43	71	RI (literature), MS
Propanoic acid	79–09-4	173,212 ± 145,233	248,943 ± 431,472	0.670570ns	1519	1517	74	73	45	74	28	45	RI (literature), MS
Octadecanoic acid, methyl ester	112–61-8	354,349 ± 377,832	275,281 ± 255,934	0.694896ns	2410	2426	74	87	83	74	87	43	RI (literature), MS
Pentadecanoic acid, methyl ester	7132-64-1	101,061 ± 110,808	78,437 ± 73,870	0.700862ns	2096	2099	74	87	143	74	87	43	RI (literature), MS
Acetic acid	64–19-7	870,872 ± 455,134	748,317 ± 687,599	0.716305ns	1428	1428	60	45	43	43	45	60	RI (literature), MS
Eicosanoic acid, methyl ester	1120-28-1	7,733 ± 10,282	8,840 ± 12,277	0.868470ns	2615	2617	74	87	45	74	87	43	RI (literature), MS
9-Octadecenoic acid, methyl ester, (E)-	1937-62-8	113,886 ± 115,338	115,450 ± 89,378	0.980333ns	2431	2445	55	69	97	55	69	74	RI (literature), MS

(a) Normalized peak area was calculated by the following formula: [{(Peak area of target compound)/(Peak area of 2-Methylheptan-3-one)}*Average peak area of 2-Methylheptan-3-one]/(Peak area of Naphthalene-d8)*(Average peak area of Naphthalene-d8) and divided by the dried cell mass (mg) after baking. (b) Statistical significance was determined using unpaired *t*-test (*, **, *** denote *p* < 0.05, *p* < 0.01 and *p* < 0.001, respectively). (c) All VOCs, except for highlighted in blue, were tentatively identified with both Mass Spec (MS) data and Retention Index (RI). Regarding the compounds shown as ‘MS, RI (standard)’ in the ‘identification’ column, RI was obtained by the authentic standard injection. RI of the other VOCs were taken from the literature which used DB-Wax column, these are considered as tentatively identified.

## 3 Discussion

In this study, we demonstrated that aroma volatiles in cultivated fat cells can be altered by media supplementation, resulting in different flavors in fat, while maintaining rapid proliferation and robust adipogenesis capability. Prior to the investigation of flavor precursor supplementation, we aimed to select comparatively better media conditions to achieve rapid cell proliferation between the three proliferation media previously reported (40, 41, 54). While bFGF has a positive effect on both proliferation and differentiation for the cells (55, 56), the cost has been highlighted as an issue (57). Therefore, as an alternative we considered the addition of small molecule compounds, as previously reported in studies with MSCs (40). An improvement in cell proliferation rate was observed with the addition of bFGF or ACY, which aligns with previous reports on pDFAT or human MSCs (Figure 1A) (40, 41). Additionally, cells maintained in ‘20%FBS + ACY’ or ‘15%FBS + bFGF’ exhibited smaller cell sizes compared to those maintained in ‘20%FBS’ (Figure 1B). Several prior studies have reported that aged cells tend to have larger sizes, whereas stem cells or those undergoing rapid self-renewal cycles are typically smaller (58, 59), consistent with the results reported here, where the cells maintained with ‘20%FBS + ACY’ or ‘15%FBS + bFGF’ showed faster proliferation and higher adipogenic capability (Figure 1D). These results showed that bFGF remains a crucial supplement for maintaining rapid cell proliferation and robust differentiation capability. Small molecule cocktails such as ACY are desirable as a substitute for bFGF, however, there was a subsequent unexpected challenge in that cells maintained with ‘20%FBS + ACY’ exhibited stronger cell adhesion, requiring over 20 min of cell dissociation treatment after the 20th passage (data not shown). The exploration of appropriate combinations and concentrations of each inhibitor could resolve this cell dissociation problem. Further, if these compounds can be replaced with food-grade materials, they could be utilized as effective growth promoters and factors for maintaining adipogenic capability, yet also keep costs lower and thus potentially improve regulatory acceptability.

In terms of adipogenic differentiation, three different adipogenic media which were previously reported were tested in the present study (42–44). Media2 contains commonly used components for inducing adipogenesis, including insulin, IBMX (isobutylmethylxanthine), rosiglitazone, and dexamethasone (Dex) (43). Media3 includes only two inducers, insulin and rosiglitazone additions to the essential minimum required for adipogenesis (44). Media1 is based on a previously reported medium (42), to which Intralipid, has been added. Media1, which contained Intralipid, demonstrated the most efficient lipid accumulation in our isolated pDFAT cells (Figure 1D). It is hypothesized that Intralipid, which contain lecithin and fatty acids, significantly enhance adipogenic differentiation (60–62). Therefore, it can be inferred that these components contribute substantially to the highest lipid accumulation among three different adipogenesis media studied in the present research. Future research should focus on the consideration of lipid-based additives that can balance the regulation of fatty acid composition with the promotion of efficient fat accumulation. Although supplementation with aroma precursors such as L-methionine inhibited cell proliferation at concentrations above 2.0 mM, it was well tolerated during the adipogenic phase and did not impair lipid accumulation (Figures 2A,B,C). This observation implies the possibility that the altered metabolic state of differentiating adipocytes confers greater tolerance to L-methionine, thereby enabling the use of higher concentrations required for efficient volatile compound production.

A tendency toward intracellular accumulation of thiamine and L-methionine upon media supplementation was observed, although the differences did not reach statistical significance (Figures 3A,B). High doses of thiamine-HCl or L-methionine raise concerns regarding the potential formation of undesirable thermal by-products—such as certain heterocyclic amines (63) or compounds generated through oxidative stress (64) —during the cooking process, rendering further safety assessment essential. Additionally, further testing of various media supplements would be desirable to achieve enhanced nutritional fortification.

Thiamine-HCl supplementation led to the statistically significant increase of 4-methyl-5-thiazoleethanol (Figure 5A), and there is a tendency for thiazole, tetrahydro-, which is predictably identified, to be induced; however, no statistically significant difference was observed (Table 2). On the other hand, thiols and thiophenes which were supposed to be derived from thiamine, were not detected in this experimental setup. Higher sensitivity detection methods, such as GC–MS/MS, could potentially confirm additional thiamine degradation products in the cooked cultivated fat supplemented with thiamine-HCl. Furthermore, it’s possible that the amount of cell sample influences the detection of sulfide-containing VOCs. Therefore, if we prepare the cell samples through suspension culture and provide a larger amount cells, such as on a gram scale, we may be able to detect such VOCs (18).

**Table 2:** All the volatile organic compounds (VOCs) found in 120 °C heated cultivated fat maintained with non-supplemented media (Control) and supplemented with 500 μM thiamine-HCl.

		Normalized Peak Area^a^ (Average ± SD)			RI^c^		m/z (actual)			m/z (NIST)			
Name	CAS#	Control	Thiamine-HCl	*p* value^b^	Actual	Ref	1	2	3	1	2	3	Identification
4-methyl-5-thiazoleethanol	504–78-9	34,801 ± 29,213	596,494 ± 149,490	0.0000009****	2268	2271	112	113	143	112	113	143	RI (standard), MS
2-Propanone, 1-hydroxy-	1638-16-0	39,789 ± 14,488	64,125 ± 16,195	0.0155*	1275	1278	43	74	42	43	31	74	RI (literature), MS
Dodecanoic acid, methyl ester	111–82-0	11,640 ± 17,303	54,750 ± 46,633	0.0433*	1788	1770	74	87	43	74	87	41	RI (literature), MS
9,12-Octadecadienoic acid, ethyl ester	7619-08-1	7,345 ± 7,247	16,766 ± 10,711	0.0863ns	2506	2515	81	67	95	67	81	55	RI (literature), MS
Benzaldehyde	100–52-7	25,877 ± 8,698	37,080 ± 12,813	0.0882ns	1487	1502	106	105	77	77	106	105	RI (literature), MS
9,12,15-Octadecatrienoic acid, methyl ester, (Z, Z, Z)-	301–00-8	63,244 ± 72,270	147,952 ± 98,005	0.1006ns	2537	2550	79	95	67	79	67	95	RI (literature), MS
Hexadecanoic acid, methyl ester	112–39-0	824,531 ± 864,907	1,662,487 ± 890,549	0.1138ns	2204	2207	74	87	143	74	87	43	RI (literature), MS
Ethyl 9-hexadecenoate	54546–22-4	6,735 ± 5,144	13,282 ± 8,501	0.1150ns	2265	2267	55	88	69	55	88	69	RI (literature), MS
9,12-Octadecadienoic acid (Z, Z)-, methyl ester	112–63-0	399,566 ± 456,548	855,335 ± 517,402	0.1194ns	2472	2488	81	67	95	67	81	95	RI (literature), MS
9-Octadecenoic acid, methyl ester, (E)-	1937-62-8	796,200 ± 866,043	1,452,439 ± 641,588	0.1548ns	2433	2445	264	265	222	264	97	96	RI (literature), MS
Thiazole, tetrahydro-	13019–20-0	3,105 ± 5,474	16,242 ± 23,483	0.1763ns	1454	1454	89	88	43	89	43	42	RI (literature), MS
Tetradecane	629–59-4	71,370 ± 17,261	95,141 ± 47,750	0.2429ns	1397	1400	57	71	43	57	43	71	RI (literature), MS
2-Decanone	513–86-0	75,638 ± 71,964	41,813 ± 26,916	0.3022ns	1478	1476	58	43	71	58	43	71	RI (literature), MS
Butylated Hydroxytoluene	128–37-0	8,811 ± 15,390	51,760 ± 112,526	0.3359ns	1891	1902	205	220	177	205	220	57	RI (literature), MS
2-Butanone, 3-hydroxy-	3658-77-3	245,097 ± 195,382	342,392 ± 173,977	0.3672ns	1264	1265	45	43	88	45	43	88	RI (literature), MS
2-Nonanone	705–86-2	75,255 ± 71,218	46,516 ± 23,726	0.3676ns	1374	1379	58	43	71	42	58	41	RI (literature), MS
2,3-Butanediol	104–61-0	23,896 ± 32,533	12,507 ± 10,666	0.4317ns	1530	1544	45	57	43	45	43	57	RI (literature), MS
Octanal	124–13-0	14,679 ± 19,686	8,008 ± 4,649	0.4372ns	1272	1277	56	57	43	43	44	56	RI (literature), MS
2-Octanone	821–55-6	25,557 ± 17,099	32,143 ± 14,852	0.4780ns	1269	1278	58	43	71	43	58	41	RI (literature), MS
2-Heptanone	693–54-9	67,854 ± 44,580	87,608 ± 54,498	0.4867ns	1173	1174	43	58	71	43	58	71	RI (literature), MS
2(3H)-Furanone, 5-ethyldihydro-	71–41-0	9,013 ± 6,490	6,922 ± 3,186	0.4893ns	1665	1665	85	57	56	85	29	56	RI (literature), MS
Furan, 2-pentyl-	3777-69-3	12,447 ± 10,782	9,907 ± 7,602	0.6391ns	1213	1215	81	82	138	81	82	132	RI (literature), MS
Dimethyl Sulfoxide	67–68-5	156,198 ± 144,743	193,834 ± 166,644	0.6711ns	1560	1553	63	78	61	63	78	45	RI (literature), MS
4-Octanone	589–63-9	3,104 ± 2,805	3,694 ± 2,823	0.7132ns	1215	1231	71	57	85	43	57	71	RI (literature), MS
Tetradecanoic acid, ethyl ester	124–06-1	5,232 ± 4,731	5,990 ± 3,893	0.7609ns	2035	2040	88	101	55	88	101	43	RI (literature), MS
Dodecane	112–40-3	7,088 ± 11,036	5,478 ± 7,491	0.7683ns	1192	1199	57	43	71	57	43	71	RI (literature), MS
2-Octenal, (E)-	111–13-7	5,506 ± 2,251	5,959 ± 3,295	0.7747ns	1409	1416	70	55	41	41	55	29	RI (literature), MS
Heptanal	111–71-7	11,392 ± 4,561	10,958 ± 4,406	0.8655ns	1174	1171	70	44	55	70	41	44	RI (literature), MS
1-Pentanol	71–41-0	11,649 ± 6,937	12,243 ± 8,824	0.8943ns	1243	1,255	42	55	70	42	55	41	RI (literature), MS
2-Pentadecanone	2548-87-0	16,462 ± 3,256	15,945 ± 11,927	0.9138ns	2002	2002	58	43	59	58	43	59	RI (literature), MS
Hexadecane	544–76-3	25,382 ± 15,849	24,715 ± 6,722	0.9256ns	1596	1600	57	71	85	57	43	71	RI (literature), MS
Nonanal	124–19-6	32,805 ± 13,222	32,214 ± 13,871	0.9388ns	1377	1379	57	56	43	57	41	43	RI (literature), MS
Pentadecane	629–62-9	12,416 ± 8,583	12,505 ± 4,808	0.9826ns	1497	1500	57	71	43	57	43	71	RI (literature), MS

(a) Normalized peak area was calculated by the following formula: [{(Peak area of target compound)/(Peak area of 2-Methylheptan-3-one)}*Average peak area of 2-Methylheptan-3-one]/(Peak area of Naphthalene-d8)*(Average peak area of Naphthalene-d8) and divided by the dried cell mass (mg) after baking. (b) Statistical significance was determined using unpaired *t*-test (*, **** denote *p* < 0.05 and *p* < 0.0001, respectively). (c) All VOCs, except for highlighted in blue, were tentatively identified with both Mass Spec (MS) data and Retention Index (RI). Regarding the compounds shown as ‘MS, RI (standard)’ in the ‘identification’ column, RI was obtained by the authentic standard injection. RI of the other VOCs were taken from the literature which used DB-Wax column, these are considered as tentatively identified.

L-methionine supplementation enhanced the production of methional, which emits a potato-like aroma (31, 32) during heating/cooking. However, it was implied that L-methionine supplementation has the possibility to promote the generation of methanethiol, which is predictably identified, a degradation product of methional (65) (Table 3). Methanethiol is described as having an onion-like odor, and at higher concentrations, it can be perceived as an unpleasant smell. To fully assess the impact of L-methionine on the aroma profile of cultivated pork fat, further studies incorporating descriptive sensory panels are required.

**Table 3:** All the volatile organic compounds (VOCs) found in 120 °C heated cultivated fat maintained with non-supplemented media (Control) and supplemented with 5 mM L-methionine.

		Normalized Peak Area^a^ (Average ± SD)			RI^c^		m/z (actual)			m/z (NIST)			
Name	CAS#	Control	L-Methionine	*p* value^b^	Actual	Ref	1	2	3	1	2	3	Identification
Propanal, 3-(methylthio)-	3268-49-3	4,740 ± 2,262	42,525 ± 16,282	0.0001***	1426	1431	104	48	76	104	48	76	RI (standard), MS
Hexanoic acid	142–62-1	48,124 ± 18,270	148,427 ± 110,493	0.0372*	1832	1825	60	73	41	60	73	41	RI (literature), MS
Methanethiol	74–93-1	3,161 ± 2,718	23,564 ± 28,747	0.0861ns	#N/A	675	47	48	45	47	48	45	MS
Butylated Hydroxytoluene	128–37-0	27,996 ± 29,442	113,047 ± 128,497	0.1160ns	1889	1902	205	220	206	205	220	57	RI (literature), MS
9,12-Octadecadienoic acid (Z, Z)-, methyl ester	112–63-0	422,566 ± 406,408	894,927 ± 748,273	0.1862ns	2478	2488	81	67	95	67	81	95	RI (literature), MS
2-Propanone, 1-hydroxy-	116–09-6	38,467 ± 7,445	50,576 ± 21,237	0.1875ns	1278	1275	43	74	42	43	31	74	RI (literature), MS
2-Heptenal, (Z)-	57266–86-1	3,061 ± 1,291	4,842 ± 3,075	0.1944ns	1306	1318	83	55	41	41	27	55	RI (literature), MS
Butanoic acid, 4-hydroxy-	591–81-1	29,963 ± 17,518	15,751 ± 17,633	0.1971ns	1586	1601	42	86	56	42	86	41	RI (literature), MS
9-Hexadecenoic acid, methyl ester, (Z)-	1120-25-8	267,204 ± 235,007	545,751 ± 464,267	0.1986ns	2230	2242	55	69	74	55	69	74	RI (literature), MS
9,12,15-Octadecatrienoic acid, methyl ester, (Z, Z, Z)-	301–00-8	55,156 ± 54,377	117,723 ± 107,237	0.2104ns	2535	2550	79	95	67	79	67	95	RI (literature), MS
2H-Pyran-2-one, tetrahydro-6-pentyl-	705–86-2	16,827 ± 11,148	29,693 ± 22,635	0.2181ns	2160	2151	99	71	70	99	71	42	RI (literature), MS
Ethyl Oleate	111–62-6	24,926 ± 27,723	100,137 ± 153,113	0.2244ns	2466	2476	55	69	88	43	55	69	RI (literature), MS
Hexadecanoic acid, methyl ester	112–39-0	1,114,934 ± 889,183	1,984,005 ± 1,493,321	0.2329ns	2206	2207	74	87	143	74	87	43	RI (literature), MS
Ethyl 9-hexadecenoate	54546–22-4	4,441 ± 2,966	21,551 ± 36,357	0.2347ns	2268	2267	55	69	88	55	86	69	RI (literature), MS
Hexadecanoic acid, ethyl ester	628–97-7	22,836 ± 22,665	96,718 ± 156,622	0.2383ns	2243	2256	88	101	157	88	101	43	RI (literature), MS
Acetamide	60–35-5	96,160 ± 48,995	157,141 ± 122,275	0.2545ns	1744	1764	59	44	43	44	59	43	RI (literature), MS
Pentadecanoic acid, ethyl ester	41114–00-5	3,334 ± 2029	7,613 ± 9,253	0.2556ns	2138	2135	88	101	43	88	101	43	RI (literature), MS
9-Octadecenoic acid, methyl ester, (E)-	1937-62-8	526,327 ± 468,695	922,417 ± 681,370	0.2576ns	2433	2445	55	97	83	55	69	74	RI (literature), MS
Octadecanoic acid, methyl ester	112–61-8	235,950 ± 234,773	446,622 ± 379,020	0.2594ns	2415	2426	74	87	43	74	87	43	RI (literature), MS
Dimethyl Sulfoxide	67–68-5	153,060 ± 103,797	315,929 ± 349,953	0.2649ns	1558	1553	63	78	61	63	78	45	RI (literature), MS
Propanoic acid	79–09-4	190,152 ± 121,252	342,095 ± 315,662	0.2668ns	1519	1517	74	73	45	74	28	45	RI (literature), MS
Heptadecanoic acid, ethyl ester	14010–23-2	4,248 ± 3,845	11,909 ± 17,407	0.2780ns	2343	2340	88	101	43	88	101	43	RI (literature), MS
Hexadecane	544–76-3	26,627 ± 9,669	34,735 ± 17,266	0.3203ns	1596	1600	57	71	43	57	43	71	RI (literature), MS
Pantolactone	599–04-2	207,027 ± 111,923	280,611 ± 131,621	0.3204ns	1990	2006	71	43	41	71	43	41	RI (literature), MS
Eicosanoic acid, methyl ester	1120-28-1	5,657 ± 6,306	12,926 ± 17,135	0.3209ns	2622	2638	74	87	43	74	87	43	RI (literature), MS
Acetic acid	64–19-7	963,578 ± 632,372	1,760,005 ± 1,975,222	0.3346ns	1427	1428	60	45	43	43	45	60	RI (literature), MS
Pentadecanoic acid, methyl ester	7132-64-1	90,538 ± 77,285	159,009 ± 164,792	0.3535ns	2096	2099	74	87	143	74	87	43	RI (literature), MS
Hexanoic acid, 2-ethyl-	149–57-5	1,035 ± 1,001	2,719 ± 4,641	0.3661ns	1952	1960	88	73	57	73	88	41	RI (literature), MS
Heptadecanoic acid, methyl ester	1731-92-6	158,197 ± 168,092	299,910 ± 367,490	0.3850ns	2306	2309	74	87	143	74	87	43	RI (literature), MS
3-Heptanol	589–82-2	5,785 ± 1,579	6,708 ± 1964	0.3873ns	1289	1290	59	69	87	59	69	41	RI (literature), MS
2-Pentadecanone	2345-28-0	11,847 ± 7,891	15,496 ± 6,539	0.4182ns	2003	2002	58	43	59	58	43	59	RI (literature), MS
Heptanal	111–71-7	11,379 ± 5,032	15,310 ± 12,138	0.4536ns	1175	1171	70	44	43	70	41	44	RI (literature), MS
Tridecane	629–50-5	7,519 ± 1,028	7,060 ± 1,008	0.4594ns	1296	1300	57	71	43	57	43	71	RI (literature), MS
Dodecanoic acid, methyl ester	111–82-0	18,191 ± 23,166	9,406 ± 12,963	0.4645ns	1786	1770	74	87	43	74	87	41	RI (literature), MS
Nonanal	124–19-6	17,176 ± 4,024	19,479 ± 7,147	0.4903ns	1378	1379	57	56	43	57	41	43	RI (literature), MS
1-Pentanol	71–41-0	12,644 ± 8,320	19,271 ± 23,045	0.4938ns	1244	1255	42	55	70	42	55	41	RI (literature), MS
Heptadecane	629–78-7	9,320 ± 6,912	12,734 ± 9,960	0.4967ns	1696	1700	57	71	85	57	43	71	RI (literature), MS
2-Heptanone	110–43-0	65,934 ± 43,249	96,537 ± 108,433	0.5090ns	1174	1178	43	58	71	43	58	27	RI (literature), MS
5-Thiazoleethanol, 4-methyl-	137–00-8	9,499 ± 13,838	4,702 ± 9,665	0.5217ns	2265	2268	112	113	143	112	113	143	RI (literature), MS
Thiazolidine, 2-methyl-	24050–16-6	2,368 ± 4,145	3,986 ± 5,137	0.5588ns	1401	1415	103	88	56	56	44	103	RI (literature), MS
Furan, 2-pentyl-	3777-69-3	14,161 ± 11,197	20,471 ± 26,571	0.5814ns	1215	1215	81	82	138	81	82	138	RI (literature), MS
2-Octanone	111–13-7	30,047 ± 20,425	38,149 ± 32,445	0.6050ns	1271	1278	43	58	71	43	58	41	RI (literature), MS
Octanal	124–13-0	27,573 ± 19,271	36,852 ± 45,405	0.6349ns	1274	1277	43	57	56	43	44	56	RI (literature), MS
2,4-Decadienal, (E, E)-	25152–84-5	6,491 ± 6,485	8,559 ± 11,632	0.7001ns	1783	1790	81	41	83	81	41	29	RI (literature), MS
Dodecane	112–40-3	7,342 ± 11,541	5,384 ± 9,312	0.7612ns	1191	1199	57	71	43	57	43	71	RI (literature), MS
Tetradecane	629–59-4	76,517 ± 15,137	74,684 ± 16,382	0.8454ns	1397	1400	57	71	43	57	43	71	RI (literature), MS
2-Decanone	693–54-9	81,570 ± 77,939	89,339 ± 120,985	0.8944ns	1478	1476	58	43	71	58	43	71	RI (literature), MS
Tetradecanoic acid, ethyl ester	124–06-1	5,048 ± 5,413	4,630 ± 5,437	0.8977ns	2039	2040	88	101	55	88	101	43	RI (literature), MS
2-Nonanone	821–55-6	79,630 ± 76,502	85,012 ± 108,250	0.9212ns	1374	1379	58	43	71	43	58	41	RI (literature), MS
Decanoic acid, methyl ester	110–42-9	18,859 ± 20,679	17,931 ± 16,058	0.9350ns	1579	1583	74	87	143	74	87	143	RI (literature), MS
Benzene, 1,3-bis(1,1-dimethylethyl)-	1014-60-4	17,327 ± 10,474	17,687 ± 18,811	0.9668ns	1411	1420	175	57	190	175	57	41	RI (literature), MS
2(3H)-Furanone, dihydro-5-pentyl-	104–61-0	88,486 ± 64,687	88,606 ± 92,242	0.9979ns	1992	1993	85	71	43	85	29	41	RI (literature), MS

(a) Normalized peak area was calculated by the following formula: [{(Peak area of target compound)/(Peak area of 2-Methylheptan-3-one)}*Average peak area of 2-Methylheptan-3-one]/(Peak area of Naphthalene-d8)*(Average peak area of Naphthalene-d8) and divided by the dried cell mass (mg) after baking. (b) Statistical significance was determined using unpaired *t*-test (*, ***denote *p* < 0.05 and *p* < 0.001, respectively). (c) All VOCs, except for highlighted in blue, were tentatively identified with both Mass Spec (MS) data and Retention Index (RI). Regarding the compounds shown as ‘MS, RI (standard)’ in the ‘identification’ column, RI was obtained by the authentic standard injection. RI of the other VOCs were taken from the literature which used DB-Wax column, these are considered as tentatively identified. The identification of Methanthiol is suspectable since it was identified by only MS.

Myoglobin in plant-based meat increases the formation of lipid oxidation products during heating, which increases the flavor complexity and is linked to characteristics such as a serum-like taste and a metallic mouthfeel (37, 66). We examined whether supplementation with myoglobin would enhance the formation of the lipid-derived aroma compounds in cultivated fat. Myoglobin significantly enhanced the formation of aldehydes, alcohols, furans, lactones and some of ketones and fatty acids (Figure 6). Notably, (E, E)-2,4-decadienal, a characteristic aroma compound known for its association with the deep-fat, meaty aroma of cooked meat, was reported to be 22 times greater than the non-supplemented condition (67) (Figure 5C). Additionally, lactones, such as *γ*-nonalactone and *δ*-decalactone, which imparts coconut-like, peachy aroma (68, 69), were each statistically significantly enhanced by 7.6 times and 12 times greater than the myoglobin-supplemented conditions. Heptanal, 1-pentanol and predictably identified aldehydes such as hexanal which exhibits fatty aroma (70, 71) also were enhanced. Hexanal has been associated with off-flavors that may be perceived as unpleasant at certain concentrations (70, 72) (Table 1). Therefore, it will be important in future studies to determine, through sensory analysis, whether its concentration reaches levels perceived as unpleasant by humans.

The results of the fatty acid analysis of the cultivated fat prior to cooking showed that the addition of myoglobin did not affect the fatty acid composition. Given the significant impact of myoglobin observed in this study, the combination of myoglobin and cultivated fat holds potential for altering aroma profiles (37, 73). Additionally, in muscle satellite cells, it has been reported that myoglobin promotes cell proliferation (74). However, considering the cytotoxicity effects of iron in cell culture (75), the post-harvest addition of myoglobin might be a more effective depending on the cell type. Fatty acid analysis revealed that cultivated fat contained 17.1% linoleic acid (Figure 4A). The decomposition of linoleic acid is known to generate aroma compounds such as (E, E)-2,4-decadienal, 2-pentylfuran and 1-pentanol, aligning with the results observed in the present study (76–79).

As part of future work, exploring the addition of supplements, such as L-glutamic acid or inosine monophosphate to enhance the umami taste, and linoleic acid rich edible oils provide further strategies to modify the flavor and nutritional profile of cultivated foods. Furthermore, investigation of masking supplements to prevent the formation of off-flavors, such as hexanal which was enhanced by myoglobin in this study is considered crucial for further enhancing desirable odor changes. Additionally, testing various combinations and concentrations of supplements could yield other insights to provoke desirable aromas. There is a possibility that both the individual effect of ribose supplementation and its combination with other flavor precursors could synergistically enhance the formation of aroma volatiles (Supplementary Figures S2, S3). Such approaches may have potential not only with porcine cells but also in cells from other tissues and other species, such as bovine (Supplementary Figure S4), as well as in muscle cells or undifferentiated (e.g., stem) cells. The present study provides evidence that the amino acid and vitamin contents, as well as the aroma volatile profiles, of cultivated fat can be systematically altered by modulating media components, thereby enhancing the potential of cultivated fat as a food ingredient.

## 4 Materials and methods

### 4.1 Cell isolation

Dedifferentiated porcine (*Sus domesticus*) cells (pDFAT) and bovine (*Bos taurus*) DFAT cells were isolated as previously described (39) from the belly (subcutaneous fat) of a 93-day-old female Yorkshire pig (DOB: 10/18/2021) and from the tailhead fat tissue (subcutaneous fat) of a 604-day-old male Angus/Holstein cross steer (DOB: 09/25/2022) were isolated. Briefly, only the modifications specific to cattle are outlined here. Adipose tissue was minced and digested in 0.2% collagenase (LS004176; Worthington Biochemical, Lakewood, NJ) dissolved in DMEM/F12 (11320033; Thermo Fisher, San Jose, CA), supplemented with 10% of antibiotic-antimycotic (100X) (15240062; Thermo Fisher) and 0.75% of 10% Pluronic F-68 (24040032; Thermo Fisher, San Jose, CA), for 1.5 h at 37 °C with shaking. The digest was filtered through 750 μm and then 300 μm cell strainers and centrifuged at 400 g for 5 min to collect mature adipocytes from the top layer of the supernatant. The lipid-rich layer was transferred to a tissue culture flask and incubated to allow stromal vascular cells to adhere, thereby separating them from the mature adipocytes. After 2 days, the floating lipids were transferred to a new tissue culture flask containing fresh media to initiate ceiling culture. Once dense colonies of lipid-laden cells were observed, the flask was flipped back to its normal position for routine maintenance.

### 4.2 Cell culture

Passage 9 pDFAT cells were thawed and cultured using three different growth media formulations which developed based on previous publications (38–41), here in after ‘20%FBS’, ‘20%FBS + ACY’ and ‘15%FBS + bFGF’ with of 0.25 μg/cm^2^ laminin 511-E8 (N-892021; Iwai North America Inc., San Carlos, CA) which added to the media during cell seeding, shown in (Supplementary Table S1). Cells were repeatedly passaged, and their doubling time compared. In ‘20%FBS + ACY’, the appropriate concentration of each inhibitor was determined by cell proliferation assay, described below (Supplementary Figure S1). Cells were maintained by passaging or stored by freezing as previously described (39). Passage 23 or 24 cells were used for GC–MS analysis, samples were seeded into 150 mm dishes (430,499; Corning, Tewksbury, MA). Passage 3 of bDFAT cells were maintained in ‘15%FBS + bFGF’ on laminin coated surface.

### 4.3 Cell proliferation assay

To determine the effect of supplementation of thiamine-HCl and L-methionine supplementation during cell proliferation, DNA amount was quantified using the CyQUANT™ Cell Proliferation Assay kit (C7026; Thermo Fisher) according to the manufacturer’s instructions. Cells were seeded in 96-well plates at 8,000 cells/cm^2^. Thiamine-HCl and L-methionine treatments were administered after the cells had adhered following seeding and cultured until the cells reach to 80% confluent. For the evaluation of A 83–01, CHIR99021 and Y-27632, cells were seeded in multiple 96-well plates at 6,000 cells/cm^2^ and cultured for 24 to 120 h. The culture medium was replaced every other day. Although cells were seeded at the same density and at the same time, each time point was measured from a separate well.

### 4.4 Adipogenic differentiation

After pDFAT cells reached 100% confluency in growth media and remained confluent for at least 24 h, their medium was replaced with adipogenic induction medium. The three different adipogenesis media formulations were developed based on (42–44) with replacing Chemically-defined FBS replacement changed to FBS, and 1% Penicillin/Streptomycin/Amphotericin (PSA) to 100 μg/mL Primocin (Supplementary Table S1). For all media compositions, the cells were fed every two days until Day 8. To assess the effect of thiamine-HCl and L-methionine, using the adipogenesis Media1, supplements were applied during the adipogenesis lipid accumulation phase, days 2 to 8, with day 0 defined as the day of media transition to adipogenesis induction media. Myoglobin treatment was limited to 24 h prior to cell harvest, due to its potential cytotoxicity and inhibition of cell proliferation (81). For each added compound, the concentration that yielded the most effective lipid accumulation was selected for use in cell culture for DHS-GC–MS analysis.

### 4.5 Lipid staining

Cultured adipocytes were stained to confirm intracellular lipid accumulation. Cells were washed twice with DPBS(−) (14190144; Thermo Fisher) to avoid cell detachment and fixed with 4% paraformaldehyde (PFA) for 20 min at room temperature (RT). After fixation, cells were first rinsed with DPBS(−), then incubated at RT for 1 h with 2 μM 4,4-difluoro-1,3,5,7,8-pentamethyl-4-bora-3a,4a-diaza-s-indacene (BODIPY 493/503, D3922; Invitrogen) diluted in DPBS(−). After BODIPY incubation, cells were rinsed three times with DPBS(−) and the cell nuclei were stained with 2 μg/mL 4′,6-diamidino-2-phenylindole (DAPI, 62247; Thermo Fisher) in DPBS(−) for 15 min at room temperature. After DAPI staining, cells were rinsed twice with DPBS(−) and stored in DPBS(−). Imaging was performed with a fluorescent widefield microscope (KEYENCE, BZ-X700, Osaka, Japan). Using this stained plate, adipogenesis efficiency was quantified by measuring normalized BODIPY intensity by Celigo Image Cytometer (200-BFFL-5C; Nexcelom Bioscience LLC, Lawrence, MA). Specifically, the average integrated intensity of BODIPY was multiplied by the area of BODIPY, and the resulting value was normalized by dividing it by the number of nuclei stained with DAPI.

### 4.6 Fat harvest

For GC–MS analysis, cells were prepared with Adipogenesis Accumulation Media 1 supplemented with supplementation of 500 μM of thiamine-HCl (T1270; Millipore Sigma, Burlington, MA) or 5.0 mM L-methionine (M5308; Millipore Sigma) or 25 mM ribose (R7500; Millipore Sigma) for the lipid accumulation phase, Day 2 to 8. 3 mg/mL of myoglobin (M0630; Millipore Sigma) was added only during the last 24 h before sample collection. The spent culture media was aspirated at Day 8, then rinsed with DPBS(−) (Thermo Fisher) 3 times. Dishes were then kept vertical for 3 min to thoroughly drain DPBS(−) and any remaining media. Once excess DPBS(−) was aspirated, the adipocytes were harvested using a cell lifter (08–100-240; Fisher Scientific), then transferred into a pre-weighed 2.0 mL tube. Samples were stored at −80 °C.

### 4.7 Metabolite analysis

Polar metabolites were extracted from frozen cell pellets using 80% methanol as previously described (80). Metabolites were separated on an Atlantis Premier BEH Z-HILIC VanGuard FIT Column: 1.7 μm, 2.1 mm × 150 mm (Waters Corporation, Milford, MA) at a flow rate of 0.175 mL/min using the following gradient: 0–20 min: linear gradient from 80–20% B; 20–20.5 min: linear gradient form 20–80% B; 20.5–28 min: hold at 80% B. Mobile Phase A was a 10 mM ammonium carbonate; Mobile Phase B was acetonitrile. To quantify thiamine and thiamine pyrophosphate, metabolites were separated on a Luna PFP(2) LC column, 3 μm, 2 mm x 100 mm (Phenomenex). Mobile Phase A was water with 0.1% formic acid; Mobile Phase B was acetonitrile, with a flow rate of 0.25 mL/min and the following gradient: 0–2 min: hold at 2% B; 2–7.5 min: linear gradient from 2–60% B; 7.5–8.5 min: linear gradient from 60–100% B; 8.5–10.5 min: hold at 100% B; 10.5–15 min: hold at 2% B. The UHPLC (Vanquish Duo; Thermo Fisher Scientific) was coupled to an Orbitrap Exploris 240 (Thermo Fisher Scientific) and full scan data were acquired in polarity switching mode at a resolution of 120,000 (m/z = 200). Relative quantitation of metabolites was performed with Skyline using a mass tolerance of 5 ppm. Compound ID was confirmed by referencing an in-house spectral library containing retention times built with authentic chemical standards.

### 4.8 Fatty acid analysis

Lipid extractions were performed using a scaled-down methyl tert-butyl ether (MTBE)-based method as described in a previous study (39). As an internal standard, 20 μL of nonadecanoic acid (N5252; Millipore Sigma) at 6 mg/mL in hexane (139386; Millipore Sigma, Burlington, MA) was added with a glass syringe. The doubly extracted MTBE phase was dried under nitrogen, saponified with 3 mL of 0.5 M sodium methoxide in methanol (92446; Millipore Sigma) at 55 °C for 30 min and then methylated with 3 mL of 14% boron trifluoride/methanol (15716; Millipore Sigma) under the same conditions. After cooling, the solution was transferred to a 15 mL polypropylene centrifuge tube, mixed with 2 mL saturated NaCl solution and 2 mL hexane (139386; Millipore Sigma), vortexed, and centrifuged at 3,500 rpm for 5 min. The upper organic phase was collected into sample vials (26590, RESTEK) for GC-FID analysis. Fatty acid composition was analyzed using an Agilent 6,890 N GC (Agilent Technologies, Santa Clara, CA) equipped with a flame ionization detector and a Select FAME capillary column (CP7430; Agilent Technologies, Santa Clara, CA; 100 m × 0.25 mm × 0.25 μm). Injection volume was 1 μL (split 1:20) at 250 °C, with helium as the carrier gas. The oven was held at 100 °C for 5 min, ramped at 10 °C/min to 220 °C (28 min), then to 250 °C (10 min). Fatty acids were identified by comparing retention times with a standard mixture (Food Industry FAME Mix, 35077; RESTEK, Bellefonte, PA). Concentrations were quantified relative to the internal standard using calibration curves of methyl nonadecanoate (74208; Sigma-Aldrich, St. Louis, MO), which showed R^2^ = 0.996 and LOD = 0.732 μg/mL (S/N > 3). Peaks with S/N < 3 were considered not detected.

## 5 Volatile compound analysis

### 5.1 Dynamic headspace GC–MS

Samples were prepared by weighing a 60 mg cell pellet into a 20 mL headspace vial (23087; RESTEK, Bellefonte, PA). As an internal standard, 1 μL of 2-Methylheptan-3-one (A284658; AmBeed, Arlington Heights, IL), prepared as 5 mg/mL in methanol, was injected into the 20 mL headspace. Additionally, prior to DHS baking, TDU tubes were loaded with Tenax® resin (11982; Millipore Sigma) and conditioned at 300 °C for 120 min under a constant flow of ultra-pure nitrogen. After conditioning, 1 μL of Naphthalene-d8 (31043; RESTEK), prepared as 10 μg/mL in dichloromethane, was injected into the Tenax® resin bed. Dichloromethane was removed by reconditioning the DHS tube at 75 °C for 5 min under a constant flow of ultra-pure nitrogen. The Naphthalene-d8 was utilized to normalize the sample injection efficiency of GC/MS, and the 2-Methylheptan-3-one was utilized to normalize the DHS extraction efficiency. The 20 mL headspace (HS) vial was transferred to the DHS module at 120 °C and incubated for 15 min. The HS vial was then purged with 200 mL ultra-pure nitrogen at 100 mL/min. The preconditioned TDU tube, packed with Tenax® resin, was then transferred to the DHS trap module at 40 °C. The HS vial was then incubated at 120 °C and purged with 1,500 mL of ultra-pure nitrogen at 50 mL/min trapping volatiles on the trap at 40 °C. The trap was then moved to the dry purge position and purged with 750 mL ultra-pure nitrogen at 100 mL/min, with the trap at 40 °C. The TDU tube was then transferred to the TDU for thermal desorption. Prior to desorption, the CIS with glass bead liner, was cooled to −120 °C with liquid nitrogen. The PTV inlet was set to a split ratio of 1:10 by selecting the solvent vent mode and setting the solvent purge to initiate at 0.01 min at 12 mL/min flow. Desorption was initiated by ramping the TDU from 40 °C to 250 °C at 720 °C/min with a 3 min hold at 250 °C. After desorption the TDU tube was removed from the TDU. The chromatographic analysis was initiated with the ramping of the CIS from −120 °C to 250 °C followed by a 3 min hold. The 7890A was outfitted with a DB-WAX UI capillary column (30 m, 0.25 mm i.d., 0.25 mm film thickness (Agilent Technologies)). The 7890A oven temperature was programmed to hold at 40 °C for 2 min; then ramp at 5 °C/min to 250 °C and then hold 250 °C for 15 min. The helium carrier gas was set to a constant flow 1.2 mL/min. The MSD was set to a solvent delay for the initial 1.25 min, with an electron energy of −70 eV, a source temperature of 250 °C, and quadrupole temperature of 150 °C. Data was acquired in scan mode ranging from 35 m/z to 450 m/z. At least three biological replicates, each with 5 to 7 injection replicates per sample, were completed.

### 5.2 GC–MS data processing

Chromatographic deconvolution was performed using PARADISe software version 6.1.7, which enabled batch processing of the full set of chromatograms. The software applies PARAFAC2 modeling within user-defined time intervals to resolve coeluting compounds. Intervals were defined to include the baseline on both sides of each peak. In cases where peaks appeared to overlap, a composite interval was created to encompass the full region as well as intervals for each visually distinct peak. Following deconvolution, the resulting mass spectra were matched against the NIST17 mass spectral libraries for “probable identification.” Only compounds with Match Quality rated as ‘Excellent’ or ‘Good’ were included. “Tentative identification” was further supported by comparison with published retention indices (RI) obtained using an identical column (DB-Wax) and chromatographic conditions. Finally, compound identities were confirmed using authentic reference standards. Deconvoluted peaks were normalized using the following formula. Deconvoluted peaks were normalized using the following formula:


$$\frac{\left\{(\frac{P_{\mathrm{TG}}}{P_{2m3h}})*P_{\mathrm{Ave}.2m3h,}\}*(P_{\mathrm{Ave}.d8N}/P_{d8N}\right)}{\text{Cell mass}\;(\mathrm{mg})}$$

P_TG_: Peak area of target compound, P_2m3h_: Peak area of 2-Methylheptan-3-one, P_Ave.2m3h_: Average peak area of 2-Methylheptan-3-one between samples, P_Ave.d8N_: Average peak area of Naphthalene-d8 between samples, P_d8N_: Peak area of Naphthalene-d8, and Cell mass represents the baked cell mass (mg) after DHS baking.

### 5.3 Volatile compounds quantification

The quantification range and the detection limit of each compound were determined by the serial dilution of the following authentic standard compounds. Each compound’s quantification range, coefficient of determination of the calibration curve, limit of detection (LOD), reagent purity of authentic standard, manufacturer, and catalog number are provided in Supplementary Table S2. In the non-supplemented control cell sample, when a peak area larger than the LOD but smaller than the limit of quantification (LOQ) was detected, the peak area was treated as the value of the LOQ, and absolute quantification was performed using the calibration curve.

## 6 Statistical analysis

Statistical analyses were performed using GraphPad Prism 10.4.1 (GraphPad Software, San Diego, CA, USA). Two-group comparisons were analyzed with unpaired *t*-tests, or with the Mann–Whitney test when sample size was limited (*n* = 3). For three or more groups, one-way ANOVA was used, followed by Tukey’s test for pairwise comparisons or Dunnett’s test when comparing to a non-supplemented control. Two-way ANOVA with multiple comparisons was applied for analyses across groups and time points. Data are shown as means ± SD, with significance set at *p* < 0.05. All experiments included at least triplicate samples (*n* ≥ 3).

## Data Availability

The original contributions presented in the study are included in the article/Supplementary material, further inquiries can be directed to the corresponding author.
