# Supplementary material for: Modulation of nutritional composition and aroma volatiles in cultivated pork fat by culture media supplementation
Source: Front Nutr. 2025 Oct 14;12:1674183. doi: 10.3389/fnut.2025.1674183 (PMC12558794; doi:10.3389/fnut.2025.1674183)
Supplement: Supplementary file 1 [file Data_Sheet_1.pdf]

Supplementary Material

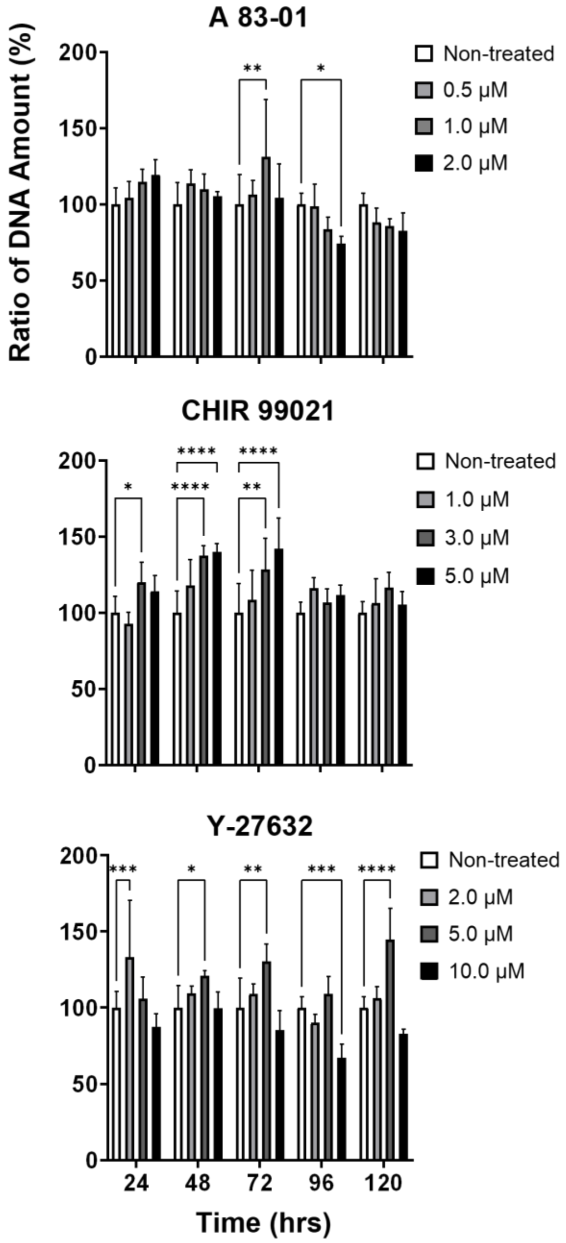

Figure S1. Effect of the three inhibitors, A 83-01, CHIR 99021 and Y-27632 (ACY) on cell proliferation to determine the optimal concentration for '20%FBS+ACY' proliferation media. From separated wells, DNA was quantified, along with the non-treated control, at each time point set to 100%. n=5 for each group. Two-way ANOVA with Dunnet's multiple comparisons was used to

calculate significant differences. (*, **, ***, **** denote P < 0.05, P < 0.01, P < 0.001 and P < 0.0001, respectively).

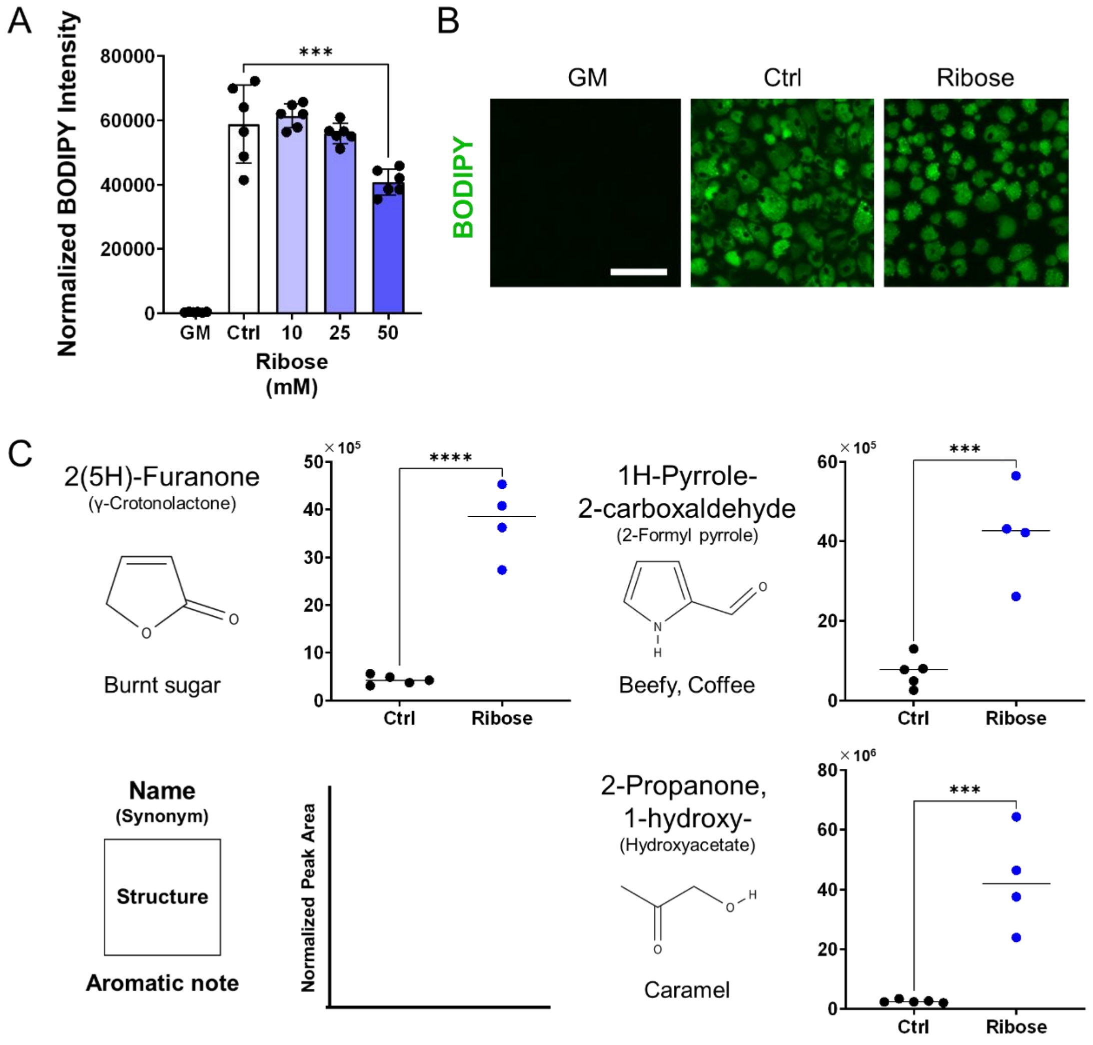

Figure S2. Effect of ribose supplementation during adipogenesis in pDFAT cells.
(A) Lipid quantification of pDFAT-derived adipocytes treated with ribose during adipogenesis lipid accumulation period. Average BODIPY integrated intensity was multiplied by BODIPY count and divided by the number of nuclei. GM; cultured in proliferation media, Ctrl; cultured in adipogenesis media without supplementation. Statistical significance was determined using one-way ANOVA followed by Dunnett's test (*** denotes P < 0.001) compared to Ctrl. **(B)** Morphology of pDFAT-derived adipocytes cultured in adipogenesis media supplemented with 25 mM Ribose, Scale bars, 100 µm. **(C)** Major volatile organic compounds (VOCs) provoked by ribose supplementations upon cell baking. VOCs were quantified by normalizing peak areas to two internal standards and normalized to cell mass after baking. 4 replicates include biological duplicates. Statistical significance was determined using unpaired t-test (***, **** denote P < 0.001 and P < 0.0001, respectively). Compounds were identified by retention index (RI) and Mass Spec referencing NIST17.

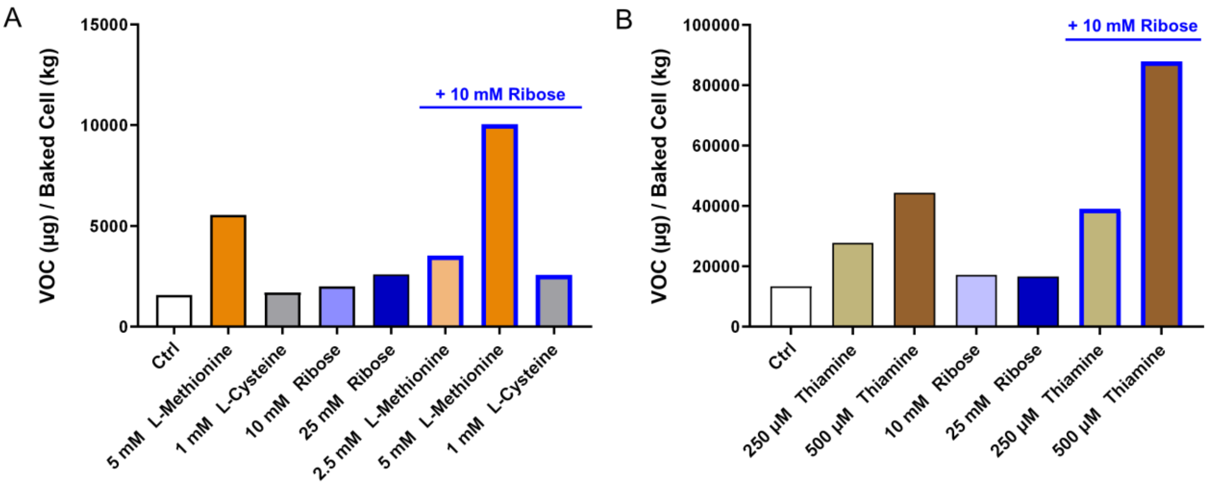

Figure S3. Quantification of **(A)** methional and **(B)** 4-methyl-5-thiazoleethanol under different media supplements and the combinations (n=1). Volatiles were quantified by normalizing peak areas to two internal standards and converting the values to mass using authentic standard curves. The resulting concentrations were further normalized to cell mass after baking. 'Ctrl' indicates the non-supplemented cell condition. Volatiles were identified by retention index (RI) and Mass Spec referencing NIST17 compared with authentic standards.

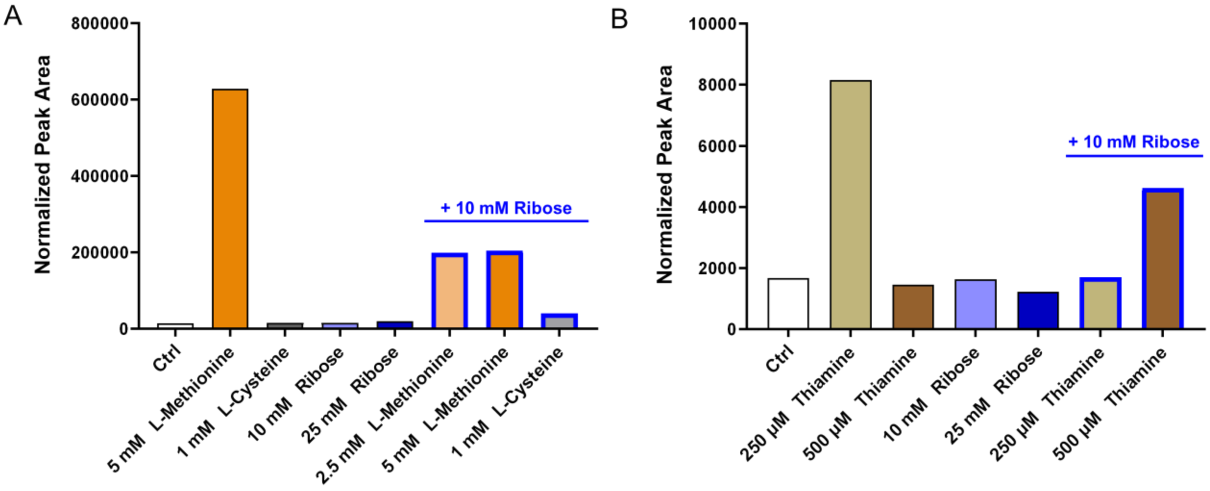

Figure S4. Quantification of **(A)** methional and **(B)** 4-methyl-5-thiazoleethanol upon baking bovine dedifferentiated fat cells (bDFAT) under media supplements and the combinations (n=1). VOCs were quantified by normalizing peak areas to two internal standards and normalized to cell mass after baking. Compounds were identified by retention index (RI) and Mass Spec referencing NIST17 compared with authentic standards.

Table S1. Cell Proliferation and Adipogenesis Media Composition

a) 3-(6-Methyl-2-pyridinyl)-N-phenyl-4-(4-quinolinyl)-1H-pyrazole-1-carbothioamide

b) 6-[[2-[[4-(2,4-Dichlorophenyl)-5-(5-methyl-1H-imidazol-2-yl)-2-pyrimidinyl]amino]ethyl]amino]-3-pyridinecarbonitrile

c) (R)-(+)-trans-4-(1-Aminoethyl)-N-(4-Pyridyl)cyclohexanecarboxamide dihydrochloride

Component	Concentration	Supplier	Catalog No.
Proliferation Media			
20% FBS			
DMEM-GlutaMax	N/A	Thermo Fisher	10569-010
Fetal Bovine Serum (FBS)	20%	Thermo Fisher	A56707-01
Primocin	100 µg/mL	InvivoGen	ant-pm-1
20%FBS + ACY			
DMEM-GlutaMax	N/A	Thermo Fisher	10569-010
FBS	20%	Thermo Fisher	A56707-01
Primocin	100 µg/mL	InvivoGen	ant-pm-1
A 83-01[a]	0.5 µM	Millipore Sigma	SML0788
CHIR99021[b]	1.0 µM	Millipore Sigma	SML1046
Y-27632[c]	2.0 µM	Millipore Sigma	Y0503
15% FBS + bFGF			
DMEM-GlutaMax	N/A	Thermo Fisher	10569-010
FBS	15%	Thermo Fisher	A56707-01
Primocin	100 µg/mL	InvivoGen	ant-pm-1
Fibroblast Growth Factor 2 (bFGF)	10 ng/mL	PeproTech	100-18B

Adipogenic Differentiation Media			
Media 1			
Induction Media (first 2 days)			
DMEM-GlutaMax	N/A	Thermo Fisher	10569-010
FBS	10%	Thermo Fisher	A56707-01
Primocin	100 µg/mL	InvivoGen	ant-pm-1
Insulin	3 µg/mL	Millipore Sigma	I0516
IBMX	0.1 mM	AdipoGen	AG-CR1-3512
Dexamethasone (Dex)	0.3 µM	Thermo Fisher	D4902
Rosiglitazone	5 µM	TCI America	R0106
Biotin	10 µM	TCI America	B0463
Calcium-D-pantothenate	5.67 uM	TCI America	P001225G
Lipid Accumulation Media (additional 6 days)			
DMEM-GlutaMax	N/A	Thermo Fisher	10569-010
Primocin	100 µg/mL	InvivoGen	ant-pm-1
Insulin	3 µg/mL	Millipore Sigma	I0516
Rosiglitazone	5 µM	TCI America	R0106
Biotin	10 µM	TCI America	B0463
L-ascorbic acid 2-phosphate trisodium salt	113 µM	FUJIFILM Wako	323-44822
Intralipid	500 µg/mL	Millipore Sigma	I141
Media 2			
Induction Media (first 3 days)			
DMEM-GlutaMax	N/A	Thermo Fisher	10569-010
FBS	1%	Thermo Fisher	A56707-01
Primocin	100 µg/mL	InvivoGen	ant-pm-1
Insulin	10 µM	Millipore Sigma	I0516
Dex	1.0 µM	Thermo Fisher	D4902
IBMX	0.5 mM	AdipoGen	AG-CR1-3512
Rosiglitazone	5 µM	TCI America	R0106
Lipid Accumulation Media (additional 6 days)			
DMEM-GlutaMax	N/A	Thermo Fisher	10569-010
FBS	1%	Thermo Fisher	A56707-01
Primocin	100 µg/mL	InvivoGen	ant-pm-1
Insulin	58.1 µg/mL	Millipore Sigma	I0516
Rosiglitazone	5 µM	TCI America	R0106
Media 3			
DMEM-GlutaMax	N/A	Thermo Fisher	10569-010
FBS	3%	Thermo Fisher	A56707-01
Primocin	100 µg/mL	InvivoGen	ant-pm-1
Insulin	10 µg/mL	Millipore Sigma	I0516
Rosiglitazone	5 µM	TCI America	R0106

Table S2. Quantification Range of Volatile Compounds. Each compound's quantification range, coefficient of determination of the calibration curve, limit of detection (LOD), reagent purity of authentic standard, manufacturer, and catalog number are described.

Compound Name	Quantification Range (µg/mL)	R^2	LOD (µg/mL)	Purity	Manufacturer	Catalog Number
4-Methyl-5-thiazoleethanol (Sulfurol)	7.81 to 500	0.997	3.910	≥98.0%	Millipore Sigma	61352
Methional	1.56 to 50	0.996	0.781	≥97%	Millipore Sigma	W274704
Furan, 2-pentyl	0.078 to 50	0.997	0.039	≥98%	Millipore Sigma	W331708
(E,E)-2,4-Decadienal	0.156 to 50	0.997	0.078	95%	Thermo Scientific	177510010
2(3H)-Furanone, dihydro-5-pentyl- (γ-Nonalactone)	0.195 to 50	0.998	0.098	≥98.5%	Millipore Sigma	44542
2H-Pyran-2-one, tetrahydro-6-pentyl- (δ-Decalactone)	0.39 to 50	0.994	0.195	≥98.0%	Millipore Sigma	74026
Heptanal	0.195 to 50	0.993	0.098	≥95%	Millipore Sigma	W254002
1-Pentanol	0.0975 to 50	0.999	0.049	≥99.8%	Millipore Sigma	77597
Benzeneacetaldehyde	0.0625 to 10	0.990	0.031	≥95%	Millipore Sigma	W287407
